# Partial pathogenicity chromosomes in *Fusarium oxysporum* are sufficient to cause disease and can be horizontally transferred

**DOI:** 10.1111/1462-2920.15095

**Published:** 2020-06-14

**Authors:** Jiming Li, Like Fokkens, Lee James Conneely, Martijn Rep

**Affiliations:** ^1^ Molecular Plant Pathology University of Amsterdam Amsterdam 1098 XH The Netherlands

## Abstract

In *Fusarium oxysporum* f.sp. *lycopersici*, all effector genes reported so far – also called *SIX* genes – are located on a single accessory chromosome which is required for pathogenicity and can also be horizontally transferred to another strain. To narrow down the minimal region required for virulence, we selected partial pathogenicity chromosome deletion strains by fluorescence‐assisted cell sorting of a strain in which the two arms of the pathogenicity chromosome were labelled with *GFP* and *RFP* respectively. By testing the virulence of these deletion mutants, we show that the complete long arm and part of the short arm of the pathogenicity chromosome are not required for virulence. In addition, we demonstrate that smaller versions of the pathogenicity chromosome can also be transferred to a non‐pathogenic strain and they are sufficient to turn the non‐pathogen into a pathogen. Surprisingly, originally non‐pathogenic strains that had received a smaller version of the pathogenicity chromosome were much more aggressive than recipients with a complete pathogenicity chromosome. Whole genome sequencing analysis revealed that partial deletions of the pathogenicity chromosome occurred mainly close to repeats, and that spontaneous duplication of sequences in accessory regions is frequent both in chromosome deletion strains and in horizontal transfer strains.

## Introduction

Accessory chromosomes, also called supernumerary chromosomes, B chromosomes, or lineage‐specific chromosomes, were first discovered in *Hemiptera* in 1907 (Camacho *et al*., 2000). However, it was only in 1991 that they were first reported in a fungus; the plant‐pathogenic fungus *Nectria haematococca* (*Fusarium solani*) (Miao *et al*., 1991). Since then, accessory chromosomes have been found in more than 20 different species of fungi (Bertazzoni *et al*., 2018), including the plant pathogens *F. oxysporum* (Fo) (Ma *et al*., 2010; Vlaardingerbroek *et al*., 2016b; Williams *et al*., 2016; van Dam *et al*., 2017), *F. solani* (Coleman *et al*., 2009) and *Zymoseptoria tritici* (Croll *et al*., 2013; Schotanus *et al*., 2015; Habig *et al*., 2017). Accessory chromosomes are generally distinguished from core chromosomes by their relatively high number of repeats, lower gene density, distinct codon usage, different evolutionary trajectories and dispensability (Galazka and Freitag, 2014).

Although accessory chromosomes are dispensable, they can play an important role under specific conditions, such as conferring pathogenicity to specific plant species (Bertazzoni *et al*., 2018). For example, in *Alternaria*, host‐selective toxin genes are located on accessory chromosomes which are responsible for causing disease on certain plant species (Tsuge *et al*., 2013; Akimitsu *et al*., 2014; Meena *et al*., 2017). Recent findings in the hemibiotrophic plant pathogen *Colletotrichum higginsianum* showed that mutants without chromosome 11 are arrested during the biotrophic phase of infection (Plaumann *et al*., 2018). In contrast, loss of this chromosome had no clear effect on vegetative fitness, suggesting that this chromosome plays a specific role during infection (Plaumann *et al*., 2018). One of the most well‐documented examples is the pathogenicity chromosome of Fo f.sp. *lycopersici* (Fol) (Ma *et al*., 2010; Schmidt *et al*., 2013; Vlaardingerbroek *et al*., 2016a). While this pathogenicity chromosome can be lost without affecting normal growth, strains without this pathogenicity chromosome do not cause disease on tomato plants (Vlaardingerbroek *et al*., 2016b). All 14 known effector genes (*Secreted In Xylem* genes, *SIX* genes) are located on this pathogenicity chromosome (Schmidt *et al*., 2013), and some of these effector genes were shown to contribute to virulence towards tomato plants, including *SIX1* (*AVR3*) (Rep *et al*., 2004), *SIX3* (*AVR2*) (Houterman *et al*., 2009) and *SIX5* (Ma *et al*., 2015). Further studies on this pathogenicity chromosome showed that loss of (most of) the long arm (q arm) of this chromosome, including *SIX6*, *SIX9* and *SIX11*, did not significantly affect virulence (Vlaardingerbroek *et al*., 2016b).

Apart from conferring advantages in a certain environment, at least some accessory chromosomes can also be horizontally transferred from one strain to another (He *et al*., 1998; Ma *et al*., 2010; Vlaardingerbroek *et al*., 2016a; van Dam *et al*., 2017). The first molecular evidence for horizontal chromosome transfer (HCT) in fungal plant pathogens was reported in *Colletotrichum gloeosporioides* (Masel *et al*., 1996). It was suggested that a 2 Mb chromosome in the biotype B isolate Bx most likely originated by a relatively recent transfer from biotype A. Shortly after, He and colleagues experimentally demonstrated horizontal transfer of a 2 Mb chromosome from biotype A to biotype B, however, no pathogenicity phenotype was transferred (He *et al*., 1998).

HCT has also been observed in Fo (Ma *et al*., 2010; Vlaardingerbroek *et al*., 2016a; van Dam *et al*., 2017; Li *et al*., 2020). When co‐incubating a Fol strain with a non‐pathogenic strain, the pathogenicity chromosome of Fol can be transferred to a non‐pathogenic strain, turning the latter into a tomato‐infecting strain (Ma *et al*., 2010; Vlaardingerbroek *et al*., 2016a). In some cases, a second accessory chromosome was co‐transferred (Ma *et al*., 2010). Similarly, from Fo f.sp. *radicis‐cucumerinum* (Forc), a single chromosome chr^RC^ can be transferred to a non‐pathogen, turning the recipient into a cucurbit‐infecting strain (van Dam *et al*., 2017). The mechanisms behind HCT are largely unknown, but it is most likely that HCT happens through heterokaryosis, which was supported by the observation that transfer is not always restricted to accessory chromosomes, but a core chromosome (~4 Mb) could also be transferred (Vlaardingerbroek *et al*., 2016a).

In a previous study, we showed that the short arm (p arm) of the pathogenicity chromosome in Fol can be sufficient for causing disease on tomato plants (Vlaardingerbroek *et al*., 2016b). In order to test this hypothesis and narrow down the genes or regions that are essential for infection in Fol, we selected partial pathogenicity chromosome deletion strains. To achieve this, we inserted the *RFP* gene in the short arm (p arm) of the pathogenicity chromosome of a strain with the *GFP* gene on the q arm (Vlaardingerbroek *et al*., 2016b), and used fluorescence‐assisted cell sorting (FACS) to select spores without GFP or RFP (Vlaardingerbroek *et al*., 2015). By testing the virulence of these deletion mutants, we show that less than half of the chromosome is sufficient for causing disease. In addition, we demonstrate that smaller versions of the pathogenicity chromosome can also be transferred to a non‐pathogenic strain, with concomitant transfer of pathogenicity towards tomato.

## Results

### Construction of a Fol strain with *GFP* and *RFP* on either arm of the pathogenicity chromosome

**Fig 1:**
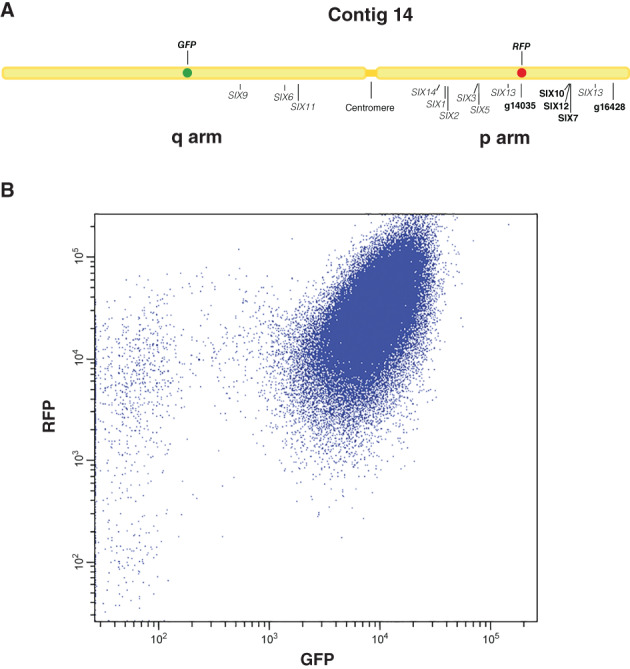
In cultures of Fol strain 14HGPR, loss of green fluorescence is much more frequent than loss of red fluorescence. A. Schematic representation of contig 14 in the new SMRT assembly of Fol4287 and genes (indicated as bold letters) selected for replacement with *RFP*. Other *Secreted In Xylem* (*SIX*) genes and the centromere are also displayed. The long arm is indicated as q arm, while the short arm is indicated as p arm. Contig 14 in the SMRT assembly of Fol4287 largely corresponds to the pathogenicity chromosome (chromosome 14 in Ma *et al*., 2010). B. Dot plot of a fluorescence assisted cell sorting experiment. Each blue dot represents a fungal spore. Most spores contain both red and green fluorescence. Some spores had lost green fluorescence, while very few spores had lost red fluorescence. Axis labels show the detection channel (*X*‐axis: *λ* = 488 nm; *Y*‐axis: *λ* = 561 nm).

To be able to select for partial pathogenicity chromosome deletion strains in Fol, we set out to create a strain with the *RFP* gene on the short arm (p arm) and the *GFP* gene on the long arm (q arm) of the pathogenicity chromosome. FACS with this strain could then be used to select spores without either green fluorescence or red fluorescence. To construct this strain, the strain 14HG6B with *GFP* on the q arm of the pathogenicity chromosome was used as a starting point (Vlaardingerbroek *et al*., 2016b). To insert the *RFP* gene on the p arm, single copy genes *FOXG_14135* and *FOXG_16428* with relatively low expression during colonization of tomato plants (van der Does *et al*., 2016) were selected for homologous recombination. With the additional purpose to investigate whether the *SIX10/12/7* gene cluster contributes to virulence, this gene cluster was also targeted for homologous recombination. The location of these genes is shown in Fig. 1A. In this study, Fol4287 was re‐sequenced with Pacbio SMRT sequencing, and contig 14 in the new SMRT assembly of Fol4287 largely corresponds to the pathogenicity chromosome (chromosome 14 in (Ma *et al*., 2010)).

To replace genes with *RFP*, *Agrobacterium*‐mediated transformation was performed. For *SIX10*/*12*/*7*, no *in locus* transformant was found after checking 433 transformants in two rounds of transformation, but two spontaneous *SIX10/12* deletion mutants, 14HG6B_ΔSIX10_12#1 and 14HG6B_ΔSIX10_12#2, were found. Since these were derived from the same experiment, it could be that they are not independent but arose from a single event. In any case, *SIX10* and *SIX12*, as part of a ~20 kb region, were lost in these strains, but *SIX7* was retained (data not shown). In the effort to replace *FOXG_16428* with *RFP*, one *in locus* transformant, 14HG6B_ΔFOXG_16428, was obtained out of 945 transformants in three rounds of transformation. However, this transformant also contained (an) ectopic insertion(s) of the *RFP* construct. For the third gene, *FOXG_14135*, 300 transformants in two rounds of transformation were checked, and we found one *in locus* transformant without ectopic insertion, 14HG6B_ΔFOXG_14135 (called **14HGPR** from hereon). Strain 14HGPR was confirmed microscopically to have both red and green fluorescence, and *in locus* insertion was confirmed by PCR using a primer just outside the flanking region in the Fol genome and another primer inside the inserted fragment (Fig. S1). Fol transformations are summarized in Table S1.

To assess the virulence of 14HGPR, bioassays were performed. Disease Index (DI) and fresh weight were scored 3 weeks after inoculation, and no significant reduction of virulence was observed when comparing 14HGPR with the original strain 14HG6B and an ectopic control (Fig. S2). Thus, 14HGPR was used for FACS experiments to obtain partial deletions of the Fol pathogenicity chromosome from both arms. In addition, bioassays were performed to assess virulence of 14HG6B_ΔSIX10/12#1, 14HG6B_ΔSIX10/12#2 and 14HG6B_ΔFOXG_16428, and no significant reduction in virulence was observed (data not shown).

### *GFP* fluorescence is much more frequently lost in spores of 14HGPR than *RFP* fluorescence

To obtain spontaneous deletions of the Fol pathogenicity chromosome from both arms, FACS of strain 14HGPR was performed to select spores without green or red fluorescence. In total, 26 different cultures were started from single spore colonies in four different FACS experiments, and from these experiments 43 *GFP* deletion strains and 18 *RFP* deletion strains were kept for further analysis (Table S2).

The first FACS experiment served to determine the approximate rate of loss of green or red fluorescence in cultures of the 14HGPR strain. Six single colonies of 14HGPR were separately inoculated into NO_3_ medium (0.17% yeast nitrogen base, 3% sucrose, 100 mM KNO_3_). After growing for 5 days, spore suspensions were obtained by filtering cultures through a double layer of mira‐cloth and directly used for FACS. We observed that all the six cultures showed a similar pattern, with a large population of spores still containing both green and red fluorescence and a very small fraction without green or red fluorescence (Fig. 1B). Strikingly, in all cultures, more spores had lost green fluorescence than spores that had lost red fluorescence, as shown in Fig. 1B. Only one single spore colony which had lost the *RFP* gene was kept for further analysis from this first experiment (Table S2).

For the second FACS experiment, 14HGPR was again mono‐spored and five single colonies were transferred directly into NO_3_ medium and allowed to grow for 5 days. This time 250 spores without green fluorescence and 226 spores without red fluorescence were deflected on Potato Dextrose Agar (PDA) plates and allowed to form colonies (Table S3). Only 27 out of 250 (11%) colonies emerged on the plates on which spores without green fluorescence were deflected, while 199 spores selected for loss of red fluorescence (87%) formed colonies (Table S3). It turned out that 18 out of the 27 (66.7%) ‘red‐selected’ colonies were confirmed to be GFP negative when checked by microscopy, but only 3 out of 199 (1.5%) ‘green‐selected’ colonies were truly RFP negative (Table S3). Since spores without red fluorescence were extremely rare, the gating was set close to spores containing both red and green fluorescence, and this apparently resulted in many false negative spores. The details from all five cultures are shown in Table S4. For the 18 ‘red’ and the 3 ‘green’ colonies, PCR was used to confirm loss of the *GFP* or *RFP* gene and other regions of the Fol pathogenicity chromosome (Table S2).

To obtain more independent deletion strains, a third FACS experiment was performed. With the aim of increasing the frequency of spontaneous loss of *RFP*, five single colonies of 14HGPR were grown on PDA plates for 10 days before collecting and inoculating spores from plates into NO_3_ medium. We suspected that the Fol pathogenicity chromosome could undergo more changes during vegetative growth on rich medium (PDA) for a relatively long time (Möller *et al*., 2018). After 5 days incubation at 25°C in the NO_3_ medium, the spores were transferred to 4°C for 5 days and 1 ml of these suspensions were transferred into new NO_3_ medium and allowed to grow for another 7 days at 25°C before being subjected to sorting. Of spores without green fluorescence deflected, 74 out of 250 (30%) formed colonies, while of spores without red fluorescence deflected, 335 out of 375 (89%) formed colonies (Table S3). Fluorescence microscopy revealed that 63 out of the 74 (85%) ‘red’ colonies were truly green fluorescence negative. However, only 10 out of 335 (3.0%) ‘green’ colonies were red fluorescence negative (Table S3). These 63 ‘red’ and 10 ‘green’ colonies were also confirmed by PCR to have lost *GFP* or *RFP* respectively. The details from all five cultures are shown in Table S5. Twenty‐four out of 63 ‘red’ and 9 out of 10 ‘green’ deletion strains were further checked for loss of other regions of the pathogenicity chromosome (Table S2). Concluding, using a longer culturing regime including incubation at 4°C, an increase in the frequency of loss of *GFP* was observed for all the five cultures, but no significant increase in the frequency of loss of *RFP* was observed when compared with the second FACS experiment (Tables [Link], [Link]).

So far, we obtained a large variety of partial deletions from the q arm of the pathogenicity chromosome, but from the p arm only 13 partial deletion strains with limited variation were found (Table S2). In a final attempt to obtain more partial deletions from the p arm of the pathogenicity chromosome, a fourth FACS experiment was performed to only select spores without red fluorescence. In this case, 10 single colonies of 14HGPR were grown on PDA plates for 1 month at 25°C, then the spores were collected from the plates and inoculated into NO_3_ medium. The cultures were incubated for 5 days in NO_3_ medium before being used for FACS. Out of 246 single colonies growing from deflected ‘green’ spores, only 5 (2%) had truly lost red fluorescence when checked microscopically (Table S3). PCR of these five single colonies confirmed that the *RFP* gene was lost in all cases (Table S2). The details from all 10 cultures are shown in Table S6.

### Illumina whole genome sequencing confirms partial deletions of the Fol pathogenicity chromosome and reveals multiplications

**Fig 2:**
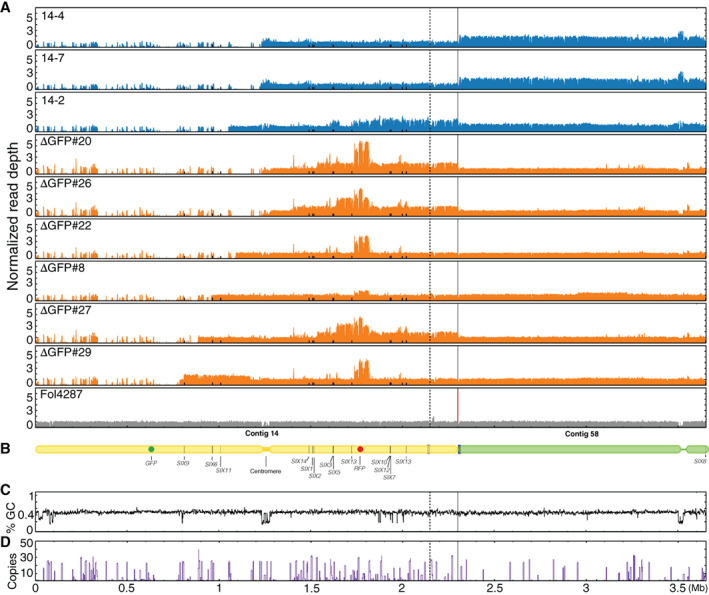
Illumina read mapping confirms partial deletions and reveals multiplications in the Fol pathogenicity chromosome in *GFP* deletion strains. A. Reads of nine *GFP* deletion strains were mapped to the SMRT assembly of Fol4287. As reference, Illumina reads of Fol4287 itself was also mapped. For comparison of differences within and between deletion strains, all genome coverage was normalized. All deletion strains had lost part of or the complete q arm of the pathogenicity chromosome. In addition, multiplications had occurred in the remaining part of the pathogenicity chromosome or contig 58 in some deletion strains. Part of contig 58 belongs to the pathogenicity chromosome as indicated between the solid and dotted lines. B. Schematic representation of the pathogenicity chromosome (contig 14 and part of contig 58) and, for comparison, the rest of contig 58. *Secreted In Xylem* (*SIX*) genes are also indicated. GC content (C) and repeat distribution across the genome (D) are also displayed.

**Fig 3:**
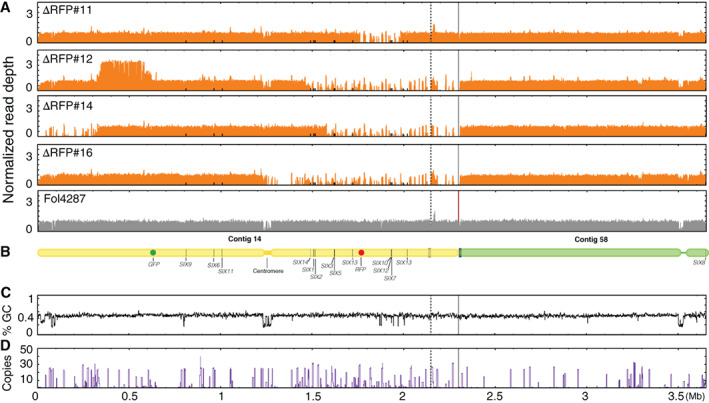
Illumina read mapping confirms partial deletions and reveals multiplications in the Fol pathogenicity chromosome in *RFP* deletion strains. A. Reads of four *RFP* deletion strains were mapped to the SMRT assembly of Fol4287. As reference, Illumina reads of Fol4287 itself was also mapped. For comparison of differences within and between deletion strains, all genome coverage was normalized. All deletion strains had lost part or complete p arm of the pathogenicity chromosome. Multiplications had occurred only in △RFP#12. Surprisingly, the end of the q arm was lost in △RFP#14. Part of the contig 58 belongs to the pathogenicity chromosome as indicated between the solid and dotted lines. B. Schematic representation of the pathogenicity chromosome (contig 14 and part of contig 58) and, for comparison, the rest of contig 58. *Secreted In Xylem* (*SIX*) genes are also indicated. GC content (C) and repeat distribution across the genome (D) are also displayed.

To more accurately assess which sequences of the pathogenicity chromosome had been lost and whether changes had also occurred in other parts of the genome, we selected 10 deletion strains with different deletion patterns for Illumina whole genome sequencing (Table S7). Of these 10 deletion strains, six strains had lost part of the q arm, and four strains had lost part of the p arm. To determine sequence changes in the deletion strains, Illumina short‐read mapping of both *GFP* deletion strains and *RFP* deletion strains was performed to the SMRT assembly of Fol4287 (Figs 2A, 3A, S3 and S4). In addition, genome sequence reads from three previously obtained deletion strains, named 14‐2, 14‐4 and 14‐7 (Vlaardingerbroek *et al*., 2016b) were mapped to the newly generated SMRT assembly of Fol4287 (Figs 2A and S3). As a reference, Fol4287 Illumina sequencing reads were retrieved from SRA and were also mapped (Figs 2A, 3A, S3A and S4A). The location of *SIX* genes is indicated as black bars (Figs 2A and 3A), and a schematic representation of the pathogenicity chromosome is shown in Figs 2B and 3B. The end of contig 14, as indicated between the solid and dashed lines in Figs 2 and 3, was originally assembled as part of contig 58. Since this part showed the same read density pattern as contig 14, in contrast to the rest of contig 58, we concluded it is part of the pathogenicity chromosome (Figs 2A and 3A). The location of the centromere is determined by assessing the GC content (Figs 2C and 3C).

All nine *GFP* deletion strains were confirmed to have lost part or complete q arm of the pathogenicity chromosome (Fig. 2A). Strains that had completely lost the q arm of the pathogenicity chromosome include 14‐4, 14‐7, △GFP#20 and △GFP#26. These deletions had happened close to or in the centromere region of the pathogenicity chromosome. Three *SIX* genes are located on the q arm, *SIX9*, *SIX6* and *SIX11*, which were lost in all these four deletion strains. Strains 14‐4 and 14‐7 were probably derived from the same deletion event since these two deletion strains showed exactly the same read density pattern (Fig. 2A). The deletion strains 14‐2 and △GFP#22 still contain a small part of the q arm, but no *SIX* genes are present in this part. The truncation of the pathogenicity chromosome in strain △GFP#8 is quite close to *SIX6*, and *SIX6* was lost in this strain (Table S2). *SIX6* and *SIX11* are present in the deletion strain △GFP#27, while *SIX9*, *SIX6* and *SIX11* are all present in △GFP#29.

In addition to partial or complete deletion of the q arm, multiplication of certain regions of the remaining part of the pathogenicity chromosome had also occurred for seven out of nine *GFP* deletion strains (Fig. 2A). For all newly obtained deletion strains in this study, multiplication had occurred in the region where *RFP* was inserted, and this probably happened during insertion of *RFP* into this location. Part of the population of △GFP#8 used to prepare genomic DNA for sequencing probably had lost the multiplication in the *RFP* region as lower read densities were observed in this region. Duplication of the q arm was only observed for △GFP#29. Except multiplication of the *RFP* region, other large multiplications of the p arm of the pathogenicity chromosome were observed for deletion strains 14‐2, △GFP#20, △GFP#26 and △GFP#27. Surprisingly, the whole contig 58 was duplicated in the deletion strains 14‐4 and 14‐7.

Lastly, to further assess whether deletions or multiplications could be linked to repeats, the distribution of repeats on the pathogenicity chromosome were determined (Fig. 2D). Except for the deletion in strain △GFP#22, the remaining eight deletions had occurred next to repeats as indicated by the high peaks (Fig. 2A and D). Among them, four deletions had occurred in the centromeric region, and the other four deletions had occurred in different locations. No large changes were observed in the core genome of the *GFP* deletion strains (Fig. S3). Interestingly, however, four out of the six newly generated deletion strains of the q arm showed the same deletion in contig 47 (Fig. S3). Additional deletions and duplications in the core genome were only observed for △GFP#29, including a relatively large deletion at the end of contig 3, a smaller deletion at the end of contig 7, and a duplication at the end of contig 61 (Fig. S3).

The four *RFP* deletion strains all showed different deletion patterns (Fig. 3A). Strain △RFP#11 had only lost a small region of the p arm of the pathogenicity chromosome, including the *SIX10*/*SIX12*/*SIX7* gene cluster. The end of the p arm of this deletion strain was still present. Strain △RFP#12 had lost a larger part of the p arm and this lost region including *SIX14*, *SIX1*, *SIX2*, *SIX3, SIX5*, *SIX13* and *SIX10*/*12*/*7*. Interestingly, △RFP#14 not only had lost part of the p arm, but it also had lost the end of the q arm. In this deletion strain, only *SIX14*, *SIX1* and *SIX2* are still present on the p arm of the pathogenicity chromosome. Complete loss of the p arm of the pathogenicity chromosome was observed for △RFP#16. In contrast to the common multiplications observed for most *GFP* deletion strains, multiplications were only observed for one *RFP* deletion strain, △RFP#12.

In the *RFP* deletion strains, all deletions and multiplications had also occurred next to repeats (Fig. 3A and D). Surprisingly, all four *RFP* deletion strains had the same deletion in contig 47 as the *GFP* deletion strains (Fig. S4). Moreover, strain △RFP#12 contains one relatively large deletion at the end of contig 2 (Fig. S4).

To conclude, partial deletions of the pathogenicity chromosome were confirmed for all the deletion strains. In addition, multiplications and additional deletions were also observed on the pathogenicity chromosome as well as other parts of the genome in some strains. Lastly, almost all deletions and multiplications had occurred in or next to repetitive regions.

### Partial Fol pathogenicity chromosomes can be transferred to a non‐pathogenic strain

To test which parts of the Fol pathogenicity chromosome can be horizontally transferred, chromosome transfer experiments were performed (van der Does and Rep, 2012) by co‐incubating each of the selected 24 deletion strains containing different partial deletions with hygromycin or zeocin‐resistant transformants of non‐pathogenic strain Fo47 (‘recipient strains’) in five independent experiments (Table S8). Since the recipient strains produced more spores than the donor strains, we decided to co‐incubate the donor strains and the recipient strains in different ratios, including 1:1, 10:1 and 20:1 (Table S9). Chromosome transfer was observed when using ratios of 1:1 or 10:1. Since the transfer frequency was extremely low, no significant difference in transfer frequency between these ratios could be determined. Co‐incubation of donor and recipient strains was performed on PDA medium or Czapek Dox Agar (CDA) in two different experiments (Table S9). Again, no significant difference in transfer frequency was observed. Since Shahi and colleagues (2016) showed that CAT medium (0.17% YNB, 25 mM KNO_3_) facilitates heterokaryon formation, which could result in HCT, we also co‐incubated the donor and recipient strains in CAT medium for 3 days before plating spores on PDA or CDA plates in one of the HCT experiments. However, no successful transfer events were observed (Table S9, HCT_IV).

Through these five experiments, we identified four strains, △GFP#8, △GFP#26, △GFP#29 and △RFP#1, with the ability to transfer their partial pathogenicity chromosome to Fo47 (Table S8). Horizontal transfer of partial chromosomes was confirmed by PCR using primers specific to the recipient strains and primers targeting different parts of the pathogenicity chromosome (Table S10). For donor strains △GFP#26 and △RFP#1, seven double drug‐resistant colonies were found for each, designated HCT_△GFP#26‐1 to ‐7 and HCT_△RFP#1‐1 to ‐7. For donor strains △GFP#8 and △GFP#29, 10 and 6 double drug‐resistant colonies were obtained, designated HCT_△GFP#8‐1 to ‐10 and HCT_△GFP#29‐1 to ‐6. Among these donor strains, △GFP#8, △GFP#26 and △GFP#29 had lost different parts of the q arm, while △RFP#1 had lost a small part of the p arm. Transfer of partial chromosomes with large deletions of the p arm were not obtained, despite several attempts (Table S8).

**Fig 4:**
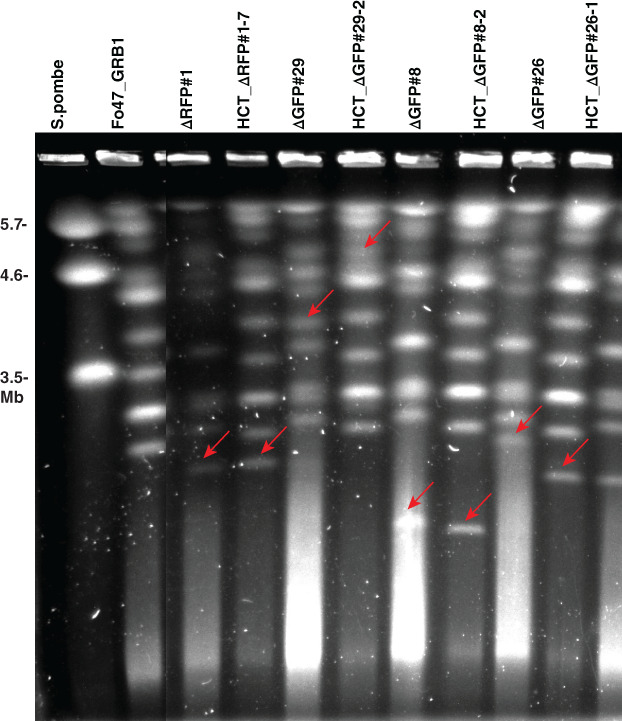
Contour‐clamped homogeneous electric field electrophoresis confirms horizontal chromosome transfer. HCT‐strains HCT_△RFP#1‐7, HCT_△GFP#29‐2, HCT_△GFP#8‐2 and HCT_△GFP#26‐1 all showed the karyotype of the recipient strain (Fo47), with an extra chromosome indicated with a red arrow. The extra chromosome in HCT_△RFP#1‐7 and HCT_△GFP#8‐2 is of a size similar to that of the partial pathogenicity chromosome in the donor strains △RFP#1 and △GFP#8 (red arrows) respectively. However, the extra chromosome in HCT_△GFP#29‐2 and HCT_△GFP#26‐1 is of a different size compared with the extra chromosome in donor strain △GFP#29 and △GFP#26 respectively (red arrows). Chromosomes of *Schizosaccharomyces pombe* was used as a marker. The figure was cropped.

It was observed earlier that chromosome size can change during HCT (Vlaardingerbroek *et al*., 2016a; van Dam *et al*., 2017). To assess karyotypes of HCT‐strains and donor strains, Contour‐clamped homogeneous electric field (CHEF) gel analysis was performed (Fig. 4). One progeny strain from each donor strain (HCT_△RFP#1‐7, HCT_△GFP#29‐2, HCT_△GFP#8‐2 and HCT_△GFP#26‐1) was selected. As expected, all HCT‐strains showed the karyotype of the recipient strain (Fo47) with an extra chromosome. For HCT_△RFP#1‐7 and HCT_△GFP#8‐2, the size of the extra chromosome is similar to the presumed partial pathogenicity chromosome in donor strains △RFP#1 and △GFP#8, respectively, which is consistent with the PCR results (Table S10) and the sequencing data (Fig. 2A). However, in donor strain △GFP#29, instead of an expected smaller version of the pathogenicity chromosome as suggested by the PCR and sequencing data (Table S10 and Fig. 2A), an extra chromosome of around 4 Mb was observed, suggesting translocation of the remaining part of the pathogenicity chromosome to another chromosome. After HCT from △GFP#29 to Fo47, an even larger chromosome (around 5 Mb) was found in the background of Fo47. In donor strain △GFP#26, which had lost the whole q arm of the pathogenicity chromosome (Fig. 2A), a larger version (~2.5 Mb) of the pathogenicity chromosome was observed, which can be explained by the multiplication of the remaining part of the pathogenicity chromosome (Fig. 2A). This ~2.5 Mb chromosome apparently became smaller after HCT (around 2 Mb in HCT_△GFP#26‐1).

### Illumina whole genome sequencing confirms transfer of partial pathogenicity chromosomes and reveals multiplications during chromosome transfer

**Fig 5:**
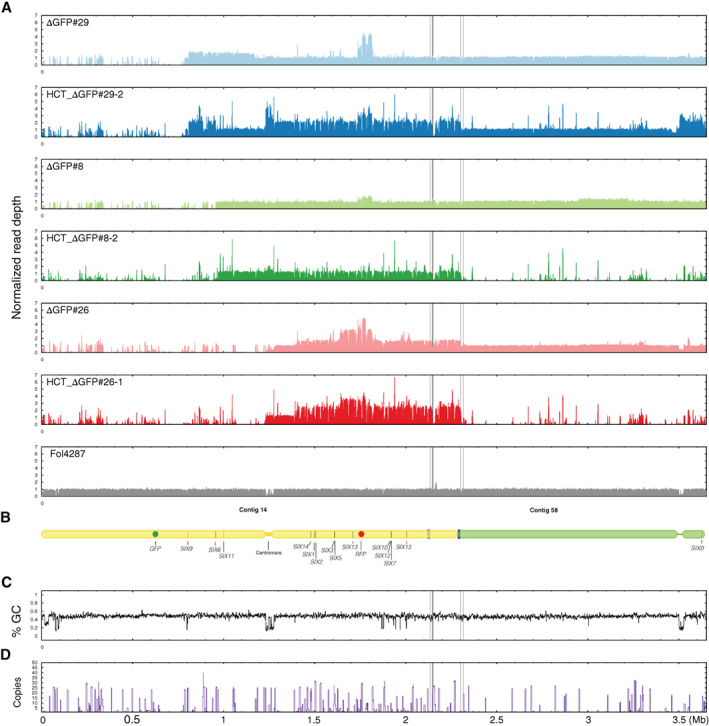
Stringent mapping of Illumina reads of HCT strains and donor strains to the SMRT assembly of Fol4287 confirms partial pathogenicity chromosome transfer. A. Illumina reads of three HCT strains (HCT_△GFP#29‐2, HCT_△GFP#8‐2 and HCT_△GFP#26‐1) and their respective donor strains (△GFP#29, △GFP#8 and △GFP#26‐1) were mapped to the SMRT assembly of Fol4287, and only those reads that mapped completely and without any mismatches were selected. As reference, Illumina reads of Fol4287 were also mapped. Partial pathogenicity chromosome transfer was confirmed for all HCT strains. Surprisingly, in HCT_△GFP#29‐2, co‐transfer contig 58 was observed while this was not observed for the other two HCT strains. In HCT_△GFP#29‐2, large multiplications of the remaining part of the pathogenicity chromosome and the end of contig 58 (not part of the pathogenicity chromosome) were also observed. Sequence multiplication during horizontal chromosome transfer was also observed in HCT_△GFP#26‐1, but not in HCT_△GFP#8‐2. B. Schematic representation of the pathogenicity chromosome (contig 14 and part of contig 58) and, for comparison, the rest of contig 58. Location of *SIX* genes and *GFP* and *RFP* are indicated. GC content (C) and repeat distribution across the genome (D) are also displayed.

To identify the sequences involved in karyotype changes (observed from the CHEF gel) during HCT, whole genomes of the HCT‐strains HCT_△GFP#29‐2, HCT_△GFP#8‐2 and HCT_△GFP#26‐1 were sequenced. To identify which sequences were newly acquired during HCT and whether core chromosomes had been transferred to HCT strains, reads of the HCT‐strains were mapped directly to the SMRT assembly of Fol4287, and only those reads that mapped completely and without any mismatches were selected (Figs 5 and S5). In the case of transfer of core genomes, a high density of perfectly mapped reads was expected, even in the subtelomeric regions. As reference, Illumina reads of donor strains were also mapped. As shown in Fig. S5, and in accordance with the karyotype patterns as observed in the CHEF gel, all HCT‐strains had the background of Fo47 with some extra sequences from the respective donor strains. Remarkably, in HCT‐strain HCT_△GFP#29‐2, the partial pathogenicity chromosome was almost fully duplicated after HCT (Fig. 5). In the same HCT strain, contig 58 and a large part of the accessory contig 47 were also co‐transferred (Figs 5 and S5), and these transferred genomic regions correspond to the duplicated region of chromosome 3/6 in the reference strain Fol4287 and the co‐transferred small chromosome in strain Fol007 (Ma *et al*., 2010). This could explain the large chromosome band observed in the CHEF gel (Fig. 4). Consistent with the CHEF gel, HCT_△GFP#8‐2 had received the partial pathogenicity chromosome from the donor strain △GFP#8, and no deletions or multiplications were observed after HCT. The partial pathogenicity chromosome from the donor strain △GFP#26 were fully transferred to the HCT‐strain HCT_△GFP#26‐1, but differences in multiplication were observed between △GFP#26 and HCT_△GFP#26‐1. These differences could explain the chromosome size difference on the CHEF gel (Fig. 4). No core chromosome transfer was observed in any HCT strain (Fig. S5).

### Single chromosome sequencing confirms partial pathogenicity chromosomes in both donor and HCT strains

**Fig 6:**
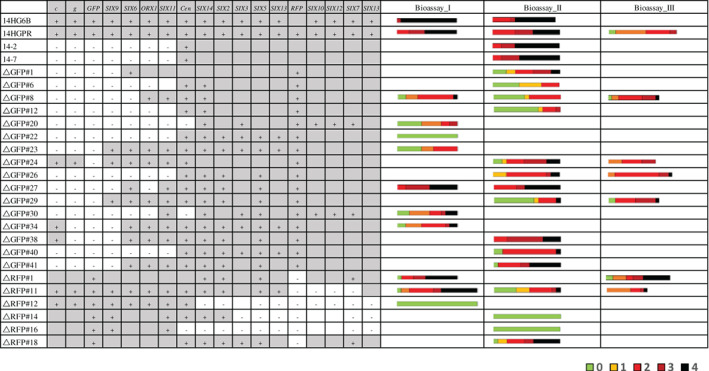
A partial pathogenicity chromosome in Fol is sufficient to cause disease on tomato plants. Bioassays were performed to assess virulence of deletion strains (+ means positive PCR result, − means negative PCR result; grey regions without symbol indicate presumed presence, white regions without symbols indicate presumed absence). Ten‐days‐old tomato seedlings were inoculated with 1 × 10^7^ spores ml^−1^ at 25°C, and DI of infected tomato plants was scored 3 weeks after inoculation. 14HGPR showed no reduced virulence compared with the parental strain 14HG6B. Deletion strains 14‐2, 14‐7 and △GFP#26, with almost complete loss of the q arm, showed no reduced virulence. Similarly, △RFP#11 without the *SIX10/12/7* gene cluster showed no reduced virulence. Deletion strains with a larger deletion of the p arm (△RFP#12, △RFP#14 and △RFP#16) did not cause disease on tomato plants. DI was scored on a scale of 0–4 (0, no symptoms; 1, one brown vessel below the cotyledons; 2, one or two brown vascular bundles at cotyledons; 3, three brown vascular bundles and growth distortion; 4, all vascular bundles are brown, plant either dead or very small and wilted). Kruskal–Wallis test was performed on disease index.

To further confirm that we correctly identified the putative pathogenicity chromosomes in the CHEF gel from the donor and HCT strains, we cut the putative pathogenicity chromosome bands from the gel and isolated DNA from the gel pieces for sequencing. In total, eight strains were selected, including three donor *GFP* deletion strains (△GFP#29, △GFP#8, △GFP#26), three HCT strains (HCT_△GFP#29, HCT_△GFP#8, HCT_△GFP#26), and two *RFP* deletion strains (△RFP#11 and △RFP#12). First, a CHEF gel was run (Fig. S6), bands were cut from the gel and 11 samples (Table S7) were sent for Illumina sequencing. Reads obtained from each sample were mapped to the SMRT assembly of Fol4287 (Fig. S7). Most bands were successfully sequenced and contained sequences from the pathogenicity chromosome, as expected (Figs 6 and S7). For example, band △GFP#8_SC in donor strain △GFP#8 and band HCT_△GFP#8_SC in the recipient strain HCT_△GFP#8 were confirmed to both contain the partial pathogenicity chromosome. Similarly, the bands in the donor strain △GFP#26 and the recipient strain HCT_△GFP#26 were also confirmed to contain the partial pathogenicity chromosome. For the third pair of donor and recipient strain, △GFP#29 and HCT_△GFP#29‐2, we observed three extra bands in the donor (△GFP#29) and two extra bands in the recipient strain (HCT_△GFP#29‐2). Therefore, five bands were cut and the isolated DNA was sequenced. Probably because of low DNA yield from the smallest band, △GFP#29_SC_XS, no reads of this sample could be mapped to the SMRT assembly. For the other two bands from the donor strain, △GFP#29_SC_L contained the partial pathogenicity chromosome (very low read densities), while △GFP#29_SC_S contained sequences from contig 7 and part of the pathogenicity chromosome. It is most likely, therefore, that the partial pathogenicity chromosome was partially duplicated and translocated to core contig 7. For the corresponding recipient strain HCT_△GFP#29‐2, the extra band HCT_△GFP#29_SC_L contained sequences of the pathogenicity chromosome as well as contig 58, and part of contig 47, and all these sequences were originated from the donor strain. This is consistent with the whole genome mapping data, which showed that these sequences were transferred (Fig. S5). We suspect that the partial pathogenicity chromosome, contig 58 and part of contig 47 formed one single chromosome that was transferred to Fo47. For the second band in the recipient strain, HCT_△GFP#29_SC_S, reads mapped abundantly to core contig 5 and much fewer reads mapped to the pathogenicity chromosome, which we suspect to be background. Finally, the bands △RFP#11_SC and △RFP#12_SC from *RFP* deletion strains △RFP#11 and △RFP#12, respectively, were confirmed to contain the expected partial pathogenicity chromosome (Fig. S7).

### Partial pathogenicity chromosomes are sufficient to cause disease on tomato

To investigate which parts of the pathogenicity chromosome of Fol are required for virulence, 22 deletion strains (Fig. 6) with different deletions in either arm of the pathogenicity chromosome were selected to assess pathogenicity. In addition, strains 14‐2 and 14‐7 obtained earlier, with large deletions in the q arm of the pathogenicity chromosome, and showing no reduced virulence on tomato in an earlier investigation, were included as controls (Vlaardingerbroek *et al*., 2016b). Again, we did not observe reduced virulence with these strains. Consistently, deletion strain △GFP#26, generated in this study and with complete loss of the q arm of the pathogenicity chromosome (Figs 2 and 6), showed no reduced virulence compared with the parental strain, 14HGPR. We conclude from this that the entire q arm is not required for virulence under the tested conditions. Strain △RFP#11, which had lost part of the p arm of the pathogenicity chromosome (Figs 3 and 6), including the *SIX10*/*12*/*7* gene cluster, also showed no reduced virulence, suggesting that this part of the chromosome is also not required for virulence. In contrast, strain △RFP#14, with a larger deletion of the p arm (Fig. 3), did not show any virulence (Fig. 6). Compared with △RFP#11, *SIX3*, *SIX5* and *SIX13* were lost in △RFP#14. Strains △RFP#12 and △RFP#16, which had lost an even larger part of the p arm, also could not cause any disease on tomato plants. Since *SIX3* and *SIX5* have been shown to contribute to contribute to virulence (Houterman *et al*., 2009; Ma *et al*., 2015), we transformed these two genes together to strain △RFP#14, but this did not lead to regaining of virulence (results not shown), indicating that *SIX13* may also be required for causing disease on tomato plants.

### Partial pathogenicity chromosomes can turn an endophyte into a pathogen

**Fig 7:**
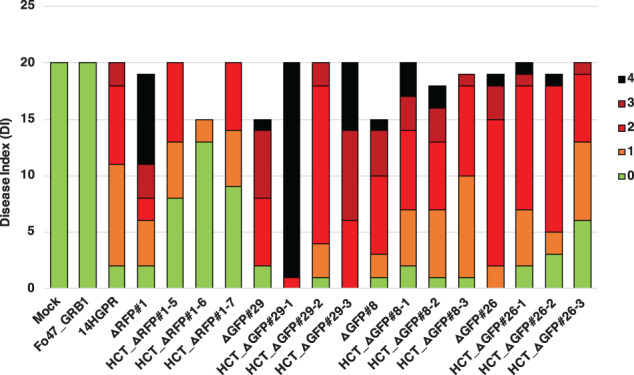
A partial pathogenicity chromosome can turn an endophyte into a pathogen. Bioassays were performed to assess the virulence of HCT strains. Ten‐days‐old tomato seedlings were inoculated with 1 × 10^7^ spores ml^−1^ at 25°C, and disease index (DI) of infected tomato plants was scored 3 weeks after inoculation. All four donor strains, △RFP#1, △GFP#29, △GFP#8 and △GFP#26 caused similar DI compared with 14HGPR. Fo47_GRB1 did not cause any disease symptoms on tomato plants, while all HCT strains in the background of Fo47_GRB1 were able to cause disease on tomato plants, with some variation in DI. DI was scored on a scale of 0–4 (0, no symptoms; 1, one brown vessel below the cotyledons; 2, one or two brown vascular bundles at cotyledons; 3, three brown vascular bundles and growth distortion; 4, all vascular bundles are brown, plant either dead or very small and wilted). Kruskal–Wallis test was performed on DI.

**Fig 8:**
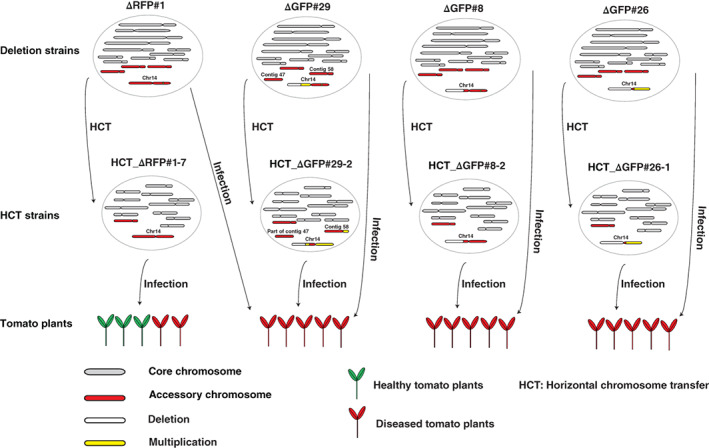
A partial pathogenicity chromosome in *Fusarium oxysporum* is sufficient to cause disease and can be horizontally transferred. Graphical summary of key observations. Strain △RFP#1 with a small deletion in the *RFP* region of the pathogenicity chromosome was still able to cause disease on tomato plants. In addition, it was able to transfer its pathogenicity chromosome to Fo47, turning the recipient strain into a tomato‐infecting strain but with only mild virulence. Deletion strain △GFP#29 had lost a large part of the long (q) arm of the pathogenicity chromosome (white bar) and underwent a duplication of the remaining part of the q arm (yellow bar). This deletion strain still caused disease on tomato plants. Surprisingly, not only the partial pathogenicity chromosome but also (the rest of) contig 58 and part of contig 47 were transferred from △GFP#29 to Fo47. These transferred sequences most likely form one big chromosome, according to single chromosome sequencing (Fig. S7) and CHEF gel analysis (Fig. S6). The resulting HCT strain HCT_△GFP#29‐2 caused severe disease on tomato plants. Deletion strain △GFP#8 had lost a large part of the q arm of the pathogenicity chromosome but it still caused disease on tomato plants. The partial pathogenicity chromosome in △GFP#8 could be transferred into Fo47, turning the recipient strain into a tomato pathogen (HCT_△GFP#8‐2) with the same virulence as its donor, △GFP#8. Deletion strain △GFP#26 had completely lost the q arm of the pathogenicity chromosome and underwent an almost complete duplication of the short arm (p arm). This version of the pathogenicity chromosome could also be transferred into Fo47. Both the donor strain △GFP#26 and the recipient strain HCT_△GFP#26‐1 caused disease on tomato plants to similar levels.

To investigate whether the endophytic strain Fo47 becomes a pathogen on tomato plants after receiving a partial Fol pathogenicity chromosome, three HCT‐strains derived from each donor strain were selected to assess their virulence. For the four donor strains, △RFP#1, △GFP#29, △GFP#8 and △GFP#26, there was no significant difference in virulence compared with the parental strain 14HGPR (Fig. 7). Fo47 could not cause any disease symptoms on tomato plants. However, all the HCT‐strains were pathogenic to tomato plants with some variations in DI. For HCT‐strains derived from △RFP#1, HCT_△RFP#1‐5, HCT_△RFP#1‐6 and HCT_△RFP#1‐7, which acquired an almost complete pathogenicity chromosome, a relatively low DI was observed (Figs 7 and 8), which is consistent with the results from previous studies (Ma *et al*., 2010; Vlaardingerbroek *et al*., 2016b). Surprisingly, for HCT_△GFP#29‐1, HCT_△GFP#29‐2 and HCT_△GFP#29‐3, much higher virulence was observed, comparable to, or even stronger than, the donor strain △GFP#29 (Fig. 7). These HCT strains had large multiplications of the remaining part of the pathogenicity chromosome as well as co‐transfer of contig 58 and part of contig 47 (Fig. 8), but these co‐transferred contigs do not contain any known *SIX* genes (Ma *et al*., 2010). Interestingly, for HCT_△GFP#8‐1, HCT_△GFP#8‐2 and HCT_△GFP#8‐3, derived from △GFP#8 and HCT_△GFP#26‐1, HCT_△GFP#26‐2 and HCT_△GFP#26‐3, derived from △GFP#26, all strains in which only a partial pathogenicity chromosome was present, higher virulence was also observed compared with HCT strains containing the complete pathogenicity chromosome (Figs 7 and 8). In these cases, no extra sequences were co‐transferred, and in △GFP#8‐derived strains no multiplication of pathogenicity chromosome sequences was observed.

## Discussion

The pathogenicity chromosome in Fol is required for causing disease on tomato plants (Ma *et al*., 2010; Plaumann *et al*., 2018), and can be horizontally transferred to a non‐pathogenic strain, turning the latter into a tomato pathogen (Ma *et al*., 2010; Vlaardingerbroek *et al*., 2016b). Here, we narrow down the regions and genes on the Fol pathogenicity chromosome that are required for virulence. Furthermore, we demonstrate that partial pathogenicity chromosomes can still be horizontally transferred to a non‐pathogenic strain, and this is sufficient to turn that strain into a pathogen. Surprisingly, transfer of a partial pathogenicity chromosome leads to higher virulence than transfer of a complete pathogenicity chromosome. Possibly, sequences in the missing (q) arm suppress virulence in the genetic background of the non‐pathogenic recipient strain, Fo47.

### How many effector genes does Fol need to cause disease on its host?

Effector genes have been predicted and studied in many different plant pathogenic fungi (Stergiopoulos and de Wit, 2009; Petre *et al*., 2014; Zhang and Xu, 2014; Lo Presti *et al*., 2015; de Wit, 2016; Selin *et al*., 2016), including *Cladosporium fulvum* (Joosten *et al*., 1997; Hammond‐Kosack *et al*., 1998; Kruger, 2002; Rooney, 2005; de Jonge *et al*., 2010; Sánchez‐Vallet *et al*., 2013), *F. oxysporum* (Rep *et al*., 2004, 2005; Houterman *et al*., 2008, 2009; van der Does *et al*., 2008; Ma *et al*., 2015), *Leptosphaeria maculans* (Gout *et al*., 2006; Van de Wouw *et al*., 2014; Ghanbarnia *et al*., 2015, 2018; Plissonneau *et al*., 2016, 2018; Petit‐Houdenot *et al*., 2019), *Magnaporthe oryzae* (Sweigard *et al*., 1995; Farman and Leong, 1998; Bryan *et al*., 2000; Yoshida *et al*., 2009; Kanzaki *et al*., 2012; Fernandez and Orth, 2018), *Melampsora lini* (Petre *et al*., 2014; Anderson *et al*., 2016; Lorrain *et al*., 2019) and *Blumeria graminis* (Bourras *et al*., 2018; Liang *et al*., 2018; Saur *et al*., 2019). In many cases, deletion of a single effector gene has little effect on virulence, suggesting functional redundancy of effectors (Selin *et al*., 2016). Nevertheless, it is likely that a limited number of effectors are required for virulence (Vlaardingerbroek *et al*., 2016b). In Fol, a single chromosome contains all effector genes (*SIX* genes) required and sufficient for causing disease on tomato (Ma *et al*., 2010; Schmidt *et al*., 2013; Vlaardingerbroek *et al*., 2016b). This provides the opportunity to study the minimal regions or genes on this chromosome that are sufficient for infection. Previously, several *SIX* genes have been shown to contribute to virulence, including *SIX1* (Rep *et al*., 2004), *SIX3* (Houterman *et al*., 2009) and *SIX5* (Ma *et al*., 2015). Moreover, Vlaardingerbroek and coworkers (Vlaardingerbroek *et al*., 2016b) have shown that most of the long (q) arm of the pathogenicity chromosome, including *SIX9*, *SIX6* and *SIX11*, is not required for virulence. Here, we confirm that the complete q arm of the pathogenicity chromosome is dispensable for pathogenicity. We further demonstrate that a large part of the p arm is also not required for virulence. This part contains *SIX10*/*12*/*7* gene cluster, indicating that the xylem secreted proteins Six7, Six10 and Six12 are not required for virulence. Deletion strains with a larger deletion of the p arm are not able to infect tomato plants, suggesting that the remaining part of the p arm of the pathogenicity chromosome is required for virulence. This region contains *SIX14*, *SIX1*, *SIX2*, *SIX3, SIX5* and *SIX13*. We conclude that, although all *SIX* genes are highly expressed during infection (van der Does *et al*., 2016) and the corresponding Six proteins are abundant in the xylem sap of infected tomato plants (Schmidt *et al*., 2013), only a subset of these proteins are required for virulence.

### Except effector genes, what else on the pathogenicity chromosome does Fol need to cause disease on tomato?

Effectors secreted by Fol during host colonization determine the outcome of Fol‐tomato interactions. The expression of effector genes is regulated by transcription factors encoded on both core and accessory chromosomes (van der Does *et al*., 2016). It has been shown that a homologue of transcription factor gene *FTF1* (FOXG_17084), which is located close to *SIX2*, can induce expression of most *SIX* genes when overexpressed (van der Does *et al*., 2016). Two additional homologues of *FTF1* are present on the pathogenicity chromosome – one is close to *SIX11* and another one is close to the *SIX10/12/7* gene cluster. Deletion strains without either of the latter two *FTF1* homologues are still fully virulent on tomato plants, indicating that they are dispensable for virulence. All deletion strains that are still virulent, however, contain the *FTF1* homologue FOXG_17084, so this homologue may be important for virulence (van der Does *et al*., 2016).

In addition, a predicted secondary metabolite gene cluster, including seven genes, is located close to the *SIX10*/*12*/*7* gene cluster (Schmidt *et al*., 2013). This cluster can also be lost without affecting virulence. Besides *SIX14*, *SIX1*, *SIX2*, *SIX3, SIX5*, *SIX13* and the *FTF1* homologue FOXG_17084, approximately 70 additional predicted protein‐coding genes reside within the part of the pathogenicity chromosome that all virulent deletion strains have in common, and some of these might contribute to virulence.

### Why are HCT strains with a partial pathogenicity chromosome more virulent than HCT strains with a complete pathogenicity chromosome?

Here, we show that HCT strains with a complete pathogenicity chromosome only caused mild virulence on tomato plants, which is consistent with previous studies (Ma *et al*., 2010; Vlaardingerbroek *et al*., 2016b). This indicates that other factors are needed for full virulence. Indeed, HCT strains with a second co‐transferred chromosome in Fol007 are more aggressive (Ma *et al*., 2010), which is also confirmed in this study. Virulence is higher in HCT_△GFP#29‐2 with co‐transferred contig 58 and part of contig 47 (which correspond to the second co‐transferred chromosome in Fol007). The increased virulence in these HCT strains may be due to the presence of transcription factor genes on the co‐transferred regions (van der Does *et al*., 2016). In addition, higher virulence was also observed for HCT_△GFP#26‐1, and duplication of almost the whole p arm could contribute to this higher virulence (Fig. 5). Surprisingly, in HCT_△GFP#8‐2 no extra sequences were co‐transferred and no multiplication was observed, but it still caused severe disease on tomato plants. It could be that sequences in the missing (q) arm suppress virulence in the genetic background of the non‐pathogenic recipient strain, Fo47. It was proposed earlier that some transcription factors encoded on the pathogenicity chromosome could suppress the expression of effector genes and maintain a saprotrophic state (van der Does *et al*., 2016). It would be interesting to investigate whether the transcription factor genes located on the missing part of the q arm indeed suppress the expression of effector genes.

### How stable is the Fol genome?

The genomes of many plant pathogenic fungi have a high level of structural variation (Johnson *et al*., 2001; Coleman *et al*., 2009; Raffaele and Kamoun, 2012; Dong *et al*., 2015; Strom and Bushley, 2016; Vanheule *et al*., 2016), including conserved core genome and lineage‐specific regions or chromosomes that are characterized by a relatively high number of repetitive elements. The core genome is generally rather stable, but the lineage‐specific regions are much more dynamic (Vlaardingerbroek *et al*., 2016b; Habig *et al*., 2017; Möller *et al*., 2018; Plaumann *et al*., 2018). In Fol strain 4287, lineage‐specific regions include four accessory chromosomes (3, 6, 14 and 15), part of core chromosome 1 and part of core chromosome 2 (Ma *et al*., 2010). The differences in stability (loss and duplication) between the core genome and the accessory genome has been investigated previously (Vlaardingerbroek *et al*., 2016b). For example, the loss frequency of lineage‐specific chromosomes was estimated to be approximately 1 in 35 000 in spores in a liquid culture. Surprisingly, core chromosome 12 in Fol4287 can also be lost but at a very low rate of 1 in 190 000 spores (Vlaardingerbroek *et al*., 2016b; Fokkens *et al*., 2018). In *Z. tritici*, spontaneous accessory chromosome loss rate was much higher with chromosome loss in 2% to > 50% of cells during 4 weeks of incubation (Möller *et al*., 2018). In the present study, we observed small deletions and duplications in core chromosomes, tending to occur at the end of chromosomes, as has also been observed in *Z. tritici* (Möller *et al*., 2018). Surprisingly, we observed recurrent deletion in contig 47 in 8 out of 10 deletion strains (Figs S3 and S4). We also observed that (part of) the pathogenicity chromosome can be lost frequently (around 6 in 100 000 spores) (Fig. 1B). It appeared that the p‐arm of the pathogenicity chromosome is more stable than the q‐arm (Fig. 1B). However, this could be explained by multiplication of the region where *RFP* was inserted in the p‐arm (Fig. 2). Presumably, this region has undergone multiplication following homologous recombination in the original strain. To conclude, in Fol, we can confirm that the core genome is rather stable except for the telomeric regions, while the accessory chromosomes are relatively dynamic.

In the present study, a large variety of deletion and multiplication patterns have been observed for the pathogenicity chromosome, and almost all the deletions or multiplications have occurred in or next to repetitive elements. It is well known that repetitive elements can lead to intra‐ or interchromosome homologous recombination, resulting in deletions or translocations (Hedges and Deininger, 2007). It has also been shown that in some fungi the facultative heterochromatin mark H3K27me3 is present in both the subtelomeric regions of the core chromosomes and accessory chromosomes (Galazka and Freitag, 2014; Fokkens *et al*., 2018). This difference in histone modification compared with the core chromosomes may play a role in the difference in chromosome stability (Fokkens *et al*., 2018; Möller *et al*., 2019).

A highly dynamic accessory genome may accelerate the evolution of the pathogen in the arms race with its host (Croll and McDonald, 2012). Many effector genes are located in the accessory part of the genome in many plant pathogenic fungi (Coleman *et al*., 2009; Ma *et al*., 2010; Peng *et al*., 2019). However, effectors can be recognized by R proteins in plants, resulting in an immune response. Mutation or loss of effector genes can help to avoid recognition and regain virulence. The accessory part of the genome may provide a niche for rapid diversification of effector genes without influencing basic cellular functions.

## Experimental procedures

### Cloning

To replace *FOXG_14135*, *FOXG_16428* or *SIX10/12/7* with *RFP*, three constructs pRW1p_Pfem1_RFP_FOXG_14135, pRW1p_Pfem1_RFP_FOXG_16428 and pRW1p_Pfem1_RFP_SIX10/12/7 were made. Each of them contains a right border (facilitating *Agrobacterium tumefaciens* mediated transformation), the flanking sequences of each gene, the *FEM1* promoter, the *RFP* open reading frame (ORF), the *SIX1* terminator, the trpC terminator, the phleomycin ORF resistance cassette, the gpd promotor, another flank of each gene, and the left border. First, pRW2h_Pfem_RFP_Tsix1 was constructed by amplifying the *RFP* ORF from pPK2‐HPH‐RFP (van der Does *et al*., 2008) using primers FP6992 (AAAtctagaATGGCCTCCTCCGAGGACG) and FP6993 (TTTagatctTTAGGCGCCGGTGGAGTGG) followed by *XbaI*‐*BglII* digestion and inserting it into the *XbaI*‐*BglII* site of pRW2h_Pfem_MCS_Tsix1 (van der Does *et al*., 2016). Then the hygromycine resistance cassette of pRW2h_Pfem_RFP_Tsix1 was replaced by the phleomycin resistance cassette of pRW1p_Pfem_MCS_Tsix1, which was modified from pRW1p (Houterman *et al*., 2008; van der Does *et al*., 2016). This resulted in pRW1p_Pfem_RFP_Tsix1. For the *FOXG_14135* deletion construct, around 1 kb flanking regions of *FOXG_14135* were amplified using primers listed in Table S11. The two fragments were introduced into pRW1p_Pfem_RFP_Tsix1 using the HiFi cloning kit [New England Biolabs (UK)]. The same method was used to make *FOXG_16428* and *SIX10/12/7* deletion constructs (Table S11). All constructs were checked by sequencing.

### Gene replacement in Fol

14HG6B was transformed via *Agrobacterium* mediated transformation (Table S1), as described previously (Takken *et al*., 2004). Transformants were monospored by pipetting 10 μl of sterile water on the emerging colony, and spreading this on a fresh PDA plate supplemented with cefotaxime and Phleomycin. After 2 days of growth at 25°C, single colonies were picked and transferred to fresh plates. From these plates, glycerol stocks were made and these are the transformants we worked with.

### Fluorescence‐assisted cell sorting

FACS was used to select independent chromosome 14 deletion strains (Vlaardingerbroek *et al*., 2015). First, 14HGPR was mono‐spored and single colonies were transferred to flasks with NO_3_ medium (0.17% yeast nitrogen base, 3% sucrose, 100 mM KNO_3_) either directly or grown on PDA plates for some time before transferring to the NO_3_ medium. After growing for 5–7 days, spore suspensions were obtained by filtering cultures through a double layer of mira‐cloth. To select spores without green or red fluorescence, 25 red (not green) and 25 green (not red) fluorescent spores were deflected on each plate and allowed to form colonies for 2–3 days at 25°C. The colonies were observed using the AMG Evos FL digital inverted microscope to confirm loss of red fluorescence or green fluorescence. Confirmed colonies were transferred to new plates and allowed to grow for at least 2 weeks before DNA extraction. To determine which parts of the pathogenicity chromosome (chromosome 14) were lost, PCR primers (Table S12) were used to scan the chromosome.

### Bioassays

To test virulence of Fol transformants, deletion strains or HCT strains on tomato (line C32), the root dip method was used (Rep *et al*., 2004). Briefly, spores were collected from 5‐day‐old cultures NO_3_ medium (0.17% yeast nitrogen base, 3% sucrose, 100 mM KNO3) by filtering through miracloth (Merck; pore size of 22–25 μm). Spores were centrifuged, resuspended in sterile MilliQ water, counted, brought to a final concentration of 1 × 10^7^ spores ml^−1^ and used for root inoculation of 10‐day‐old tomato seedlings. The seedlings were then potted individually and kept at 25°C. Three weeks after inoculation, plant weight above the cotyledons was measured, and the extent of browning of vessels in the remaining part of the stem was scored. DI was scored on a scale of 0–4 (0, no symptoms; 1, one brown vessel below the cotyledons; 2, one or two brown vascular bundles at cotyledons; 3, three brown vascular bundles and growth distortion; 4, all vascular bundles are brown, plant either dead or very small and wilted).

### Horizontal chromosome transfer

To test whether partial pathogenicity chromosomes can be transferred or not, HCT experiments were performed (van der Does and Rep, 2012). In total, 24 deletion strains (Table S8) were selected to co‐incubate with Fo47pGRB1 (Vlaardingerbroek *et al*., 2016a) or Fo47‐H1 (Ma *et al*., 2010). Strains were grown in minimal liquid medium (3% sucrose, 0.17% yeast nitrogen base and 100 mM KNO_3_) for 3–5 days, after which 10^5^ or 2 × 10^5^ microconidia from the donor and recipient strains were mixed in different ratios and co‐incubated on PDA or CDA plates for 8 days. Spores were collected from these plates using 2–5 ml sterile MilliQ, filtered through sterile miracloth and pipetted on a double selective PDA plate containing 0.1 M Tris pH 8 supplemented with 100 μg ml^−1^ hygromycin (Duchefa) and 100 μg ml^−1^ zeocin (InvivoGen). Double drug resistant colonies were selected after 3 days and monospored on a new plate supplemented with both drugs. After 2–3 days of growth, colonies were selected and transferred to new plates supplemented with zeocin and hygromycin. Fluorescence of double drug‐resistant colonies was checked with an AMG Evos FL digital inverted microscope. Strains with both red and green fluorescence were allowed to grow for 2 weeks before DNA isolation. Both selection markers and other genes (Table S10) were used to confirm HCT by PCR.

### CHEF electrophoresis

To confirm HCT, CHEF electrophoresis was performed. Preparation of protoplasts and pulsed‐field gel electrophoresis were performed as described previously (Ma *et al*., 2010). *Fusarium* strains were cultured in 100 ml NO_3_ medium (0.17% yeast nitrogen base, 100 mM KNO_3_ and 3% sucrose) for 5 days at 25°C. Then, conidia were collected by filtering through a double‐layer of miracloth and the concentration of spores were measured. 5 × 10^8^ conidia were transferred to 40 ml PDB (BD biosciences). After approximately 16 h of growth at 25°C, germinated spores were re‐suspended in 10 ml MgSO_4_ solution (1.2 M MgSO_4_, 50 mM sodium citrate, pH 5.8) supplemented with 50 mg ml^−1^ Glucanex (Sigma) + 5 mg ml^−1^ driselase (Sigma, D9515) and incubated for approximately 17 h at 30°C in a shaking water bath (65 r.p.m.). The protoplasts were filtered through a double layer of miracloth, collected by centrifugation and casted in InCert agarose (Lonza) at a concentration of 2 × 10^8^ protoplasts ml^−1^. Plugs were treated with 2 mg ml^−1^ pronase E at 50°C. Chromosomes were separated by pulsed‐field electrophoresis for 260 h in 1% Seakem Gold agarose (Lonza) at 1.5 V cm^−1^ in a CHEF‐DRII system (Bio‐Rad) in 0.5 × TBE at 4°C, with switch times between 1200 and 4800 s. The gels were stained with 1 μg ml^−1^ ethidium bromide in 0.5 × TBE.

### Single chromosomes recovery from a CHEF gel

Chromosome DNA recovery from CHEF gels were performed according to the method described previously (Kashiwa *et al*., 2017). Chromosome bands of interest were excised from the gel and were placed in 2 ml microcentrifuge tubes, then heated at 100°C while shaking at 350 r.p.m. for at least 10 min to melt the agarose. After the melting step, six units of thermostable β‐agarase (Nippon gene, Tokyo, Japan) were added to the gel solution, and held at 57°C, 350 r.p.m. for 15 min. After enzyme treatment, tubes were kept on ice for 15 min to confirm the agarose was completely digested. If remaining agarose was observed in the reaction mixture, melting (100°C for 10 min) and subsequent steps were repeated. The concentration of DNA in the reaction mixture was checked by a Qubit 3.0 fluorometer (Invitrogen, Carlsbad, CA) and the Qubit dsDNA HS Assay kit (Invitrogen).

### Illumina single chromosome and whole genome sequencing

Genomic DNA isolation was performed on freeze‐dried mycelium ground in liquid nitrogen as starting material, using multiple rounds of phenol‐chloroform extraction and precipitation, as well as the Purelink plant total DNA purification kit (Invitrogen).

Illumina sequencing (150 bp paired‐end, insert size ~500 bp) was performed on a HiSeq 2500 machine at the Hartwig Medical Foundation (Amsterdam, the Netherlands) at ~100X coverage, resulting in 5.0–5.6 Mb of sequence data per sample.

Raw reads were trimmed to remove low‐quality bases and adapter sequences using fastq‐mcf v1.04.807 (‐q 20). PCR duplicates were removed using PicardTools MarkDuplicates v2.7.1 with standard settings.

To assess partial deletions of the pathogenicity chromosome, reads of deletion strains were mapped directly to the SMRT assembly of Fol4287.

Reads from single chromosomes were also mapped directly to the SMRT assembly of Fol4287.

To confirm HCT, trimmed reads were directly mapped to SMRT assembly of Fol4287, and only reads that mapped once with 100% coverage and 100% identity were selected (with SAMtools view ‐q 42) when calculating read densities.

For visualization of the reads counts in 10 kb non‐overlapping sliding windows, SAMtools bedcov was used. SAMtools version 1.8 was used in all above‐mentioned cases.

## Conflict of interest

The authors declare that they have no competing interests.

## Supporting information


**Fig S1.** *In locus* insertion of *RFP* confirmed by PCR.
To confirm homologous recombination, a primer just outside the flanking region in the Fol genome and another primer inside the inserted fragment were used for both left and right flanking regions. Ectopic insertion(s) of T‐DNA was also assessed using a primer outside the flanking region in the construct and another primer inside of the flanking region. Loss of *FOXG_14035* was confirmed by using a primer between the two flanks in the Fol genome and another primer in one flanking region. The *in locus* transformant without ectopic insertion(s) is outlined with a green rectangle. NC: negative control; 14HG6B: original strain; Construct: *FOXG_14135* deletion construct; 14HGPR: *in locus* transformant; Ectopic#1, #2: ectopic transformants.

**Fig S2.** Deletion of *FOXG_14135* does not result in reduced virulence.
Fresh weight (A) and disease index (DI) (B) of infected tomato plants were scored 3 weeks after inoculation. When 10 days old tomato seedlings were inoculated with 1 × 10^7^ spores ml^−1^ at 25°C, the *FOXG_14135* deletion strain 14HGPR showed similar disease index and fresh weight as the original strain 14HG6B. As control, disease symptoms of an ectopic transformant 14HG6B#ect (T‐DNA was randomly inserted in the genome) were assessed and no significant difference in virulence was observed compared with 14HG6B. Water (Mock)‐treated plants were completely healthy. Disease index was scored on a scale of 0–4 (0, no symptoms; 1, one brown vessel below the cotyledons; 2, one or two brown vascular bundles at cotyledons; 3, three brown vascular bundles and growth distortion; 4, all vascular bundles are brown, plant either dead or very small and wilted). One‐way ANOVA was performed on fresh weight. Kruskal–Wallis test was performed on disease index.

**Fig S3.** Illumina whole genome read mapping of *GFP* deletion strains reveals a few minor changes in the core genome.
(A) Whole genome reads of nine *GFP* deletion strains were mapped to the SMRT assembly of Fol4287. As reference, Illumina reads of Fol4287 itself were also mapped. For comparison of differences within and between deletion strains, genome coverage was normalized. No obvious changes were observed in the core genome of the three previously generated strains 14‐4, 14‐7 and 14‐2. For all the deletion strains generated in this study, the same small deletion was observed at the end of contig 0. In addition, △GFP#20, △GFP#22, △GFP#8 and △GFP#29 all showed the same deletion in contig 47. For △GFP#29, deletions at the end of contig 3 and contig 7 and one duplication at the end of contig 61 were also observed. GC content (B) and repeat distribution across the genome (C) are also displayed.

**Fig S4.** Illumina whole genome read mapping of *RFP* deletion strains reveals a few minor changes in the core genome.
(A) Whole genome reads of four *RFP* deletion strains were mapped to the SMRT assembly of Fol4287. As reference, Illumina reads of Fol4287 itself were also mapped. For comparison of differences within and between deletion strains, genome coverage was normalized. For all the deletion strains, the same small deletion was observed at the end of contig 0 and in the middle of contig 47. For △RFP#12, a deletion at the end of contig 2 was observed. GC content (B) and repeat distribution across the genome (C) are also displayed.

**Fig S5.** Stringent selection of mapped Illumina reads of HCT strains and donor strains to the SMRT assembly of Fol4287 shows absence of core chromosome transfer and confirms transfer of accessory regions.
(A) Illumina reads of HCT strains (HCT_△GFP#29‐2, HCT_△GFP#8‐2 and HCT_△GFP#26‐1) and their respective donor strains (△GFP#29, △GFP#8 and △GFP#26‐1) were mapped to the SMRT assembly of Fol4287, and only those reads that mapped completely and without any mismatches were selected. In the case of transfer of core chromosomes, a high density of perfectly mapped reads was expected, even in the subtelomeric regions as shown for the reference donor strains. No core chromosome transfer was observed for any HCT strain. (Partial) pathogenicity chromosome transfer was confirmed for all HCT strains. In HCT_△GFP#29‐2, co‐transfer of contig 58 and part of contig 47 was observed. GC content (B) and repeat distribution across the genome (C) are also displayed.

**Fig S6.** Single chromosomes cut from a CHEF gel for sequencing.
Eight strains were selected for chromosome separation in a CHEF gel. In total, 11 bands were cut from the gel (1 to 11) and sent for sequencing. The numbers are indicated on the respective bands. The same number indicates corresponding bands from the same strain. The name used in the main text for each number is listed below the gel. Chromosomes of *S. cerevisiae* were used as marker.

**Fig S7.** Single chromosome sequencing confirms partial pathogenicity chromosomes in both donor and HCT strains.
(A) Illumina reads of 11 bands cut from a CHEF gel (see Fig. S5) were mapped to the SMRT assembly of Fol4287. As reference, Illumina reads of Fol4287 itself were also mapped. Except band △GFP#29_SC_XS, which was not successfully sequenced, the remaining 10 bands indeed contained sequences of the pathogenicity chromosome (contig 14). For example, the same partial pathogenicity chromosome is present in donor strains (△GFP#8 and △GFP#26) and the respective recipient strains (HCT_△GFP#8‐2 and HCT_△GFP#26‐1). Band △GFP#29_SC_S contained sequences from contig 7 and part of the pathogenicity chromosome. Instead of band HCT_△GFP#29_SC_S, band HCT_△GFP#29_SC_L was confirmed to be the transferred chromosome, containing sequences of the pathogenicity chromosome as well as contig 58 and part of contig 47. GC content (B) and repeat distribution across the genome (C) are also displayed.

**Table S1.** Summary of *Agrobacterium*‐mediated *Fusarium* transformations.

**Table S2.** Fol pathogenicity chromosome deletion strains obtained.
Symbols used in the table: + for positive PCR result, − for negative PCR result; grey regions without symbol for presumed presence, white regions without symbols for presumed absence.

**Table S3.** Summary of three fluorescence assisted cell sorting (FACS) experiments.

**Table S4.** Details of the second fluorescence assisted cell sorting (FACS) experiment.

**Table S5.** Details of the third fluorescence assisted cell sorting (FACS) experiment.

**Table S6.** Details of the fourth fluorescence assisted cell sorting (FACS) experiment.

**Table S7.** Strains and single chromosomes sent for sequencing.

**Table S8.** Summary of Horizontal Chromosome Transfer (HCT) experiments.
No: no successful transfer; Yes: successful transfer. Only strains for which transfer was attempted are shown in this table.

**Table S9.** Different strain ratios and media used in five HCT experiments.
PDA: potato dextrose agar; CDA: Czapek Dox Agar. CAT medium: 0.17% YNB, 25 mM KNO_3._

**Table S10.** Horizontal chromosome transfer was confirmed by PCR.
Symbols used in the table: + for positive PCR result, − for negative PCR result; black regions without symbol are presumed to be present, white regions without symbols are presumed to be absent.

**Table S11.** Primers used for cloning.

**Table S12.** Markers on the pathogenicity chromosome.

## References

[emi15095-bib-0001] Akimitsu, K. , Tsuge, T. , Kodama, M. , Yamamoto, M. , and Otani, H. (2014) Alternaria host‐selective toxins: determinant factors of plant disease. J Gen Plant Pathol 80: 109–122.

[emi15095-bib-0002] Anderson, C. , Khan, M.A. , Catanzariti, A.‐M. , Jack, C.A. , Nemri, A. , Lawrence, G.J. , *et al* (2016) Genome analysis and avirulence gene cloning using a high‐density RADseq linkage map of the flax rust fungus, *Melampsora lini* . BMC Genomics 17: 667. 27550217 10.1186/s12864-016-3011-9 PMC4994203

[emi15095-bib-0003] Bertazzoni, S. , Williams, A.H. , Jones, D.A. , Syme, R.A. , Tan, K.‐C. , and Hane, J.K. (2018) Accessories make the outfit: accessory chromosomes and other dispensable DNA regions in plant‐pathogenic fungi. Mol Plant Microbe Interact 31: 779–788. 29664319 10.1094/MPMI-06-17-0135-FI

[emi15095-bib-0004] Bourras, S. , Praz, C.R. , Spanu, P.D. , and Keller, B. (2018) Cereal powdery mildew effectors: a complex toolbox for an obligate pathogen. Curr Opin Microbiol 46: 26–33. 29455142 10.1016/j.mib.2018.01.018

[emi15095-bib-0005] Bryan, G.T. , Wu, K.‐S. , Farrall, L. , Jia, Y. , Hershey, H.P. , McAdams, S.A. , *et al* (2000) A single amino acid difference distinguishes resistant and susceptible alleles of the Rice blast resistance gene pi‐ta. Plant Cell 12: 2033–2045. 11090207 10.1105/tpc.12.11.2033 PMC150156

[emi15095-bib-0006] Camacho, J.P.M. , Sharbel, T.F. , and Beukeboom, L.W. (2000) B‐chromosome evolution. Philos Trans R Soc B Biol Sci, B‐chromosome evolution 355: 163–178. 10.1098/rstb.2000.0556 PMC1692730 10724453

[emi15095-bib-0007] Coleman, J.J. , Rounsley, S.D. , Rodriguez‐Carres, M. , Kuo, A. , Wasmann, C.C. , Grimwood, J. , *et al* (2009) The genome of *Nectria haematococca*: contribution of supernumerary chromosomes to gene expansion. PLoS Genet 5: e1000618. 19714214 10.1371/journal.pgen.1000618 PMC2725324

[emi15095-bib-0008] Croll, D. , and McDonald, B.A. (2012) The accessory genome as a cradle for adaptive evolution in pathogens. PLoS Pathog 8: e1002608. 22570606 10.1371/journal.ppat.1002608 PMC3343108

[emi15095-bib-0009] Croll, D. , Zala, M. , and McDonald, B.A. (2013) Breakage‐fusion‐bridge cycles and large insertions contribute to the rapid evolution of accessory chromosomes in a fungal pathogen. PLoS Genet 9: e1003567. 23785303 10.1371/journal.pgen.1003567 PMC3681731

[emi15095-bib-0010] de Jonge, R. , Peter van Esse, H. , Kombrink, A. , Shinya, T. , Desaki, Y. , Bours, R. , *et al* (2010) Conserved fungal LysM effector Ecp6 prevents chitin‐triggered immunity in plants. Science 329: 953–955. 20724636 10.1126/science.1190859

[emi15095-bib-0011] de Wit, P.J.G.M. (2016) *Cladosporium fulvum* effectors: weapons in the arms race with tomato. Annu Rev Phytopathol 54: 1–23. 27215970 10.1146/annurev-phyto-011516-040249

[emi15095-bib-0012] Dong, S. , Raffaele, S. , and Kamoun, S. (2015) The two‐speed genomes of filamentous pathogens: waltz with plants. Curr Opin Genet Dev 35: 57–65. 26451981 10.1016/j.gde.2015.09.001

[emi15095-bib-0013] Farman, M.L. , and Leong, S.A. (1998) Chromosome walking to the AVR1‐CO39 avirulence gene of *Magnaporthe grisea*: discrepancy between the physical and genetic maps. Genetics 150: 1049. 9799257 10.1093/genetics/150.3.1049 PMC1460382

[emi15095-bib-0014] Fernandez, J. , and Orth, K. (2018) Rise of a cereal killer: the biology of *Magnaporthe oryzae* biotrophic growth. Trends Microbiol 26: 582–597. 29395728 10.1016/j.tim.2017.12.007 PMC6003838

[emi15095-bib-0015] Fokkens, L. , Shahi, S. , Connolly, L.R. , Stam, R. , Schmidt, S.M. , Smith, K.M. , *et al* (2018) The multi‐speed genome of *Fusarium oxysporum* reveals association of histone modifications with sequence divergence and footprints of past horizontal chromosome transfer events. bioRxiv. 10.1101/465070.

[emi15095-bib-0016] Galazka, J.M. and Freitag, M. (2014) Variability of chromosome structure in pathogenic fungi – of ‘ends and odds.’ Curr Opin Microbiol 20: 19–26, Variability of chromosome structure in pathogenic fungi – of ‘ends and odds’. 24835423 10.1016/j.mib.2014.04.002 PMC4133287

[emi15095-bib-0017] Ghanbarnia, K. , Fudal, I. , Larkan, N.J. , Links, M.G. , Balesdent, M.‐H. , Profotova, B. , *et al* (2015) Rapid identification of the *Leptosphaeria maculans* avirulence gene AvrLm2 using an intraspecific comparative genomics approach. Mol Plant Pathol 16: 699–709. 25492575 10.1111/mpp.12228 PMC6638346

[emi15095-bib-0018] Ghanbarnia, K. , Ma, L. , Larkan, N.J. , Haddadi, P. , Fernando, W.G.D. , and Borhan, M.H. (2018) *Leptosphaeria maculans* AvrLm9: a new player in the game of hide and seek with AvrLm4‐7. Mol Plant Pathol 19: 1754–1764. 29330918 10.1111/mpp.12658 PMC6638032

[emi15095-bib-0019] Gout, L. , Fudal, I. , Kuhn, M.‐L. , Blaise, F. , Eckert, M. , Cattolico, L. , *et al* (2006) Lost in the middle of nowhere: the AvrLm1 avirulence gene of the Dothideomycete *Leptosphaeria maculans* . Mol Microbiol 60: 67–80. 16556221 10.1111/j.1365-2958.2006.05076.x

[emi15095-bib-0020] Habig, M. , Quade, J. , and Stukenbrock, E.H. (2017) Forward genetics approach reveals host genotype‐dependent importance of accessory chromosomes in the fungal wheat pathogen *Zymoseptoria tritici* . MBio 8: e01919. 29184021 10.1128/mBio.01919-17 PMC5705923

[emi15095-bib-0021] Hammond‐Kosack, K.E. , Tang, S. , Harrison, K. , and Jones, J.D.G. (1998) The tomato Cf‐9 disease resistance gene functions in tobacco and potato to confer responsiveness to the fungal Avirulence gene product Avr9. Plant Cell 10: 1251–1266. 9707527 10.1105/tpc.10.8.1251 PMC144066

[emi15095-bib-0022] He, C. , Rusu, A.G. , Poplawski, A.M. , Irwin, J.A.G. , and Manners, J.M. (1998) Transfer of a supernumerary chromosome between vegetatively incompatible biotypes of the fungus *Colletotrichum gloeosporioides* . Genetics 150: 1459–1466. 9832523 10.1093/genetics/150.4.1459 PMC1460434

[emi15095-bib-0023] Hedges, D.J. , and Deininger, P.L. (2007) Inviting instability: transposable elements, double‐strand breaks, and the maintenance of genome integrity. Mutat Res Mol Mech Mutagen 616: 46–59. 10.1016/j.mrfmmm.2006.11.021 PMC1850990 17157332

[emi15095-bib-0024] Houterman, P.M. , Cornelissen, B.J.C. , and Rep, M. (2008) Suppression of plant resistance gene‐based immunity by a fungal effector. PLoS Pathog 4: e1000061. 18464895 10.1371/journal.ppat.1000061 PMC2330162

[emi15095-bib-0025] Houterman, P.M. , Ma, L. , van Ooijen, G. , de Vroomen, M.J. , Cornelissen, B.J.C. , Takken, F.L.W. , and Rep, M. (2009) The effector protein Avr2 of the xylem‐colonizing fungus *Fusarium oxysporum* activates the tomato resistance protein I‐2 intracellularly. Plant J 58: 970–978. 19228334 10.1111/j.1365-313X.2009.03838.x

[emi15095-bib-0026] Johnson, L.J. , Johnson, R.D. , Akamatsu, H. , Salamiah, A. , Otani, H. , Kohmoto, K. , and Kodama, M. (2001) Spontaneous loss of a conditionally dispensable chromosome from the *Alternaria alternata* apple pathotype leads to loss of toxin production and pathogenicity. Curr Genet 40: 65–72. 11570518 10.1007/s002940100233

[emi15095-bib-0027] van Dam, P. , Fokkens, L. , Ayukawa, Y. , van der Gragt, M. , ter Horst, A. , Brankovics, B. , *et al* (2017) A mobile pathogenicity chromosome in *Fusarium oxysporum* for infection of multiple cucurbit species. Sci Rep 7: 9042. 28831051 10.1038/s41598-017-07995-y PMC5567276

[emi15095-bib-0028] van der Does, H.C. , Duyvesteijn, R.G.E. , Goltstein, P.M. , van Schie, C.C.N. , Manders, E.M.M. , Cornelissen, B.J.C. , and Rep, M. (2008) Expression of effector gene SIX1 of *Fusarium oxysporum* requires living plant cells. Fungal Genet Biol 45: 1257–1264. 18606236 10.1016/j.fgb.2008.06.002

[emi15095-bib-0029] van der Does, H.C. , Fokkens, L. , Yang, A. , Schmidt, S.M. , Langereis, L. , Lukasiewicz, J.M. , *et al* (2016) Transcription factors encoded on core and accessory chromosomes of *Fusarium oxysporum* induce expression of effector genes. PLoS Genet 12: e1006401. 27855160 10.1371/journal.pgen.1006401 PMC5140021

[emi15095-bib-0030] van der Does, H.C. , and Rep, M. (2012) Horizontal transfer of supernumerary chromosomes in fungi. Methods Mol Biol 835: 427–437. 22183669 10.1007/978-1-61779-501-5_26

[emi15095-bib-0031] Joosten, M.H.A.J. , Vogelsang, R. , Cozijnsen, T.J. , Verberne, M.C. , and De Wit, P.J.G.M. (1997) The biotrophic fungus *Cladosporium fulvum* circumvents Cf‐4‐mediated resistance by producing unstable AVR4 elicitors. Plant Cell 9: 367–379. 9090881 10.1105/tpc.9.3.367 PMC156924

[emi15095-bib-0032] Kanzaki, H. , Yoshida, K. , Saitoh, H. , Fujisaki, K. , Hirabuchi, A. , Alaux, L. , *et al* (2012) Arms race co‐evolution of *Magnaporthe oryzae* AVR‐Pik and rice Pik genes driven by their physical interactions. Plant J 72: 894–907. 22805093 10.1111/j.1365-313X.2012.05110.x

[emi15095-bib-0033] Kashiwa, T. , Kozaki, T. , Ishii, K. , Turgeon, B.G. , Teraoka, T. , Komatsu, K. , and Arie, T. (2017) Sequencing of individual chromosomes of plant pathogenic *Fusarium oxysporum* . Fungal Genet Biol 98: 46–51. 27919652 10.1016/j.fgb.2016.12.001

[emi15095-bib-0034] Kruger, J. (2002) A tomato cysteine protease required for Cf‐2‐dependent disease resistance and suppression of autonecrosis. Science 296: 744–747. 11976458 10.1126/science.1069288

[emi15095-bib-0035] Li, J.‐M. , Fokkens, L. , van Dam, P. , and Rep, M. (2020) Related mobile pathogenicity chromosomes in *Fusarium oxysporum* determine host range on cucurbits. Mol Plant Pathol 21: 761. 32246740 10.1111/mpp.12927 PMC7214479

[emi15095-bib-0036] Liang, P. , Liu, S. , Xu, F. , Jiang, S. , Yan, J. , He, Q. , *et al* (2018) Powdery mildews are characterized by contracted carbohydrate metabolism and diverse effectors to adapt to obligate biotrophic lifestyle. Front Microbiol 9: 3160. 30619222 10.3389/fmicb.2018.03160 PMC6305591

[emi15095-bib-0037] Lorrain, C. , Gonçalves dos Santos, K.C. , Germain, H. , Hecker, A. , and Duplessis, S. (2019) Advances in understanding obligate biotrophy in rust fungi. New Phytol 222: 1190–1206. 30554421 10.1111/nph.15641

[emi15095-bib-0038] Ma, L.‐J. , van der Does, H.C. , Borkovich, K.A. , Coleman, J.J. , Daboussi, M.‐J. , Di Pietro, A. , *et al* (2010) Comparative genomics reveals mobile pathogenicity chromosomes in fusarium. Nature 464: 367–373. 20237561 10.1038/nature08850 PMC3048781

[emi15095-bib-0039] Ma, L. , Houterman, P.M. , Gawehns, F. , Cao, L. , Sillo, F. , Richter, H. , *et al* (2015) The AVR2‐SIX5 gene pair is required to activate I‐2 ‐mediated immunity in tomato. New Phytol 208: 507–518. 25967461 10.1111/nph.13455

[emi15095-bib-0040] Masel, A.M. , He, C. , Poplawski, A.M. , Irwin, J.A.G. , and Manners, J.M. (1996) Molecular evidence for chromosome transfer between biotypes of *Colletotrichum gloeosporioides* . Mol Plant Microbe Interact 9: 5.

[emi15095-bib-0041] Meena, M. , Gupta, S.K. , Swapnil, P. , Zehra, A. , Dubey, M.K. , and Upadhyay, R.S. (2017) Alternaria toxins: potential virulence factors and genes related to pathogenesis. Front Microbiol 8: 1451. 28848500 10.3389/fmicb.2017.01451 PMC5550700

[emi15095-bib-0042] Miao, V.P. , Covert, S.F. , and Vanetten, H.D. (1991) A fungal gene for antibiotic resistance on a dispensable (“B”) chromosome. Science 254: 1773. 1763326 10.1126/science.1763326

[emi15095-bib-0043] Möller, M. , Habig, M. , Freitag, M. , and Stukenbrock, E.H. (2018) Extraordinary genome instability and widespread chromosome rearrangements during vegetative growth. Genetics 210: 517–529. 30072376 10.1534/genetics.118.301050 PMC6216587

[emi15095-bib-0044] Möller, M. , Schotanus, K. , Soyer, J.L. , Haueisen, J. , Happ, K. , Stralucke, M. , *et al* (2019) Destabilization of chromosome structure by histone H3 lysine 27 methylation. PLos Genet 15: e1008093. 31009462 10.1371/journal.pgen.1008093 PMC6510446

[emi15095-bib-0045] Peng, Z. , Oliveira‐Garcia, E. , Lin, G. , Hu, Y. , Dalby, M. , Migeon, P. , *et al* (2019) Effector gene reshuffling involves dispensable mini‐chromosomes in the wheat blast fungus. PLoS Genet 15: e1008272. 31513573 10.1371/journal.pgen.1008272 PMC6741851

[emi15095-bib-0046] Petit‐Houdenot, Y. , Degrave, A. , Meyer, M. , Blaise, F. , Ollivier, B. , Marais, C. , *et al* (2019) A two genes – for – one gene interaction between *Leptosphaeria maculans* and *Brassica napus* . New Phytol 223: 397–411. 30802965 10.1111/nph.15762

[emi15095-bib-0047] Petre, B. , Joly, D.L. , and Duplessis, S. (2014) Effector proteins of rust fungi. Front Plant Sci 5: 416. 25191335 10.3389/fpls.2014.00416 PMC4139122

[emi15095-bib-0048] Plaumann, P.‐L. , Schmidpeter, J. , Dahl, M. , Taher, L. , and Koch, C. (2018) A dispensable chromosome is required for virulence in the Hemibiotrophic plant pathogen *Colletotrichum higginsianum* . Front Microbiol 9: 1005. 29867895 10.3389/fmicb.2018.01005 PMC5968395

[emi15095-bib-0049] Plissonneau, C. , Daverdin, G. , Ollivier, B. , Blaise, F. , Degrave, A. , Fudal, I. , *et al* (2016) A game of hide and seek between avirulence genes AvrLm4‐7 and AvrLm3 in *Leptosphaeria maculans* . New Phytol 209: 1613–1624. 26592855 10.1111/nph.13736

[emi15095-bib-0050] Plissonneau, C. , Rouxel, T. , Chèvre, A.‐M. , Van De Wouw, A.P. , and Balesdent, M.‐H. (2018) One gene‐one name: the AvrLmJ1 avirulence gene of *Leptosphaeria maculans* is AvrLm5. Mol Plant Pathol 19: 1012–1016. 28661570 10.1111/mpp.12574 PMC6638039

[emi15095-bib-0051] Lo Presti, L. , Lanver, D. , Schweizer, G. , Tanaka, S. , Liang, L. , Tollot, M. , *et al* (2015) Fungal effectors and plant susceptibility. Annu Rev Plant Biol 66: 513–545. 25923844 10.1146/annurev-arplant-043014-114623

[emi15095-bib-0052] Raffaele, S. , and Kamoun, S. (2012) Genome evolution in filamentous plant pathogens: why bigger can be better. Nat Rev Microbiol 10: 417–430. 22565130 10.1038/nrmicro2790

[emi15095-bib-0053] Rep, M. , Van Der Does, H.C. , Meijer, M. , Van Wijk, R. , Houterman, P.M. , Dekker, H.L. , *et al* (2004) A small, cysteine‐rich protein secreted by *Fusarium oxysporum* during colonization of xylem vessels is required for I‐3‐mediated resistance in tomato. Mol Microbiol 53: 1373–1383. 15387816 10.1111/j.1365-2958.2004.04177.x

[emi15095-bib-0054] Rep, M. , Meijer, M. , Houterman, P.M. , van der Does, H.C. , and Cornelissen, B.J.C. (2005) *Fusarium oxysporum* evades I‐3‐mediated resistance without altering the matching Avirulence gene. Mol Plant Microbe Interact 18: 15–23. 15672814 10.1094/MPMI-18-0015

[emi15095-bib-0055] Rooney, H.C.E. (2005) Cladosporium Avr2 inhibits tomato Rcr3 protease required for Cf‐2‐dependent disease resistance. Science 308: 1783–1786. 15845874 10.1126/science.1111404

[emi15095-bib-0056] Sánchez‐Vallet, A. , Saleem‐Batcha, R. , Kombrink, A. , Hansen, G. , Valkenburg, D.‐J. , Thomma, B.P. , and Mesters, J.R. (2013) Fungal effector Ecp6 outcompetes host immune receptor for chitin binding through intrachain LysM dimerization. Elife 2: 1. 10.7554/eLife.00790 PMC3700227 23840930

[emi15095-bib-0057] Saur, I.M.L. , Bauer, S. , Kracher, B. , Lu, X. , Franzeskakis, L. , Müller, M.C. , *et al* (2019) Multiple pairs of allelic MLA immune receptor‐powdery mildew AVRA effectors argue for a direct recognition mechanism. Elife 8: e44471. 30777147 10.7554/eLife.44471 PMC6414202

[emi15095-bib-0058] Schmidt, S.M. , Houterman, P.M. , Schreiver, I. , Ma, L. , Amyotte, S. , Chellappan, B. , *et al* (2013) MITEs in the promoters of effector genes allow prediction of novel virulence genes in *Fusarium oxysporum* . BMC Genomics 14: 119. 23432788 10.1186/1471-2164-14-119 PMC3599309

[emi15095-bib-0059] Schotanus, K. , Soyer, J.L. , Connolly, L.R. , Grandaubert, J. , Happel, P. , Smith, K.M. , *et al* (2015) Histone modifications rather than the novel regional centromeres of *Zymoseptoria tritici* distinguish core and accessory chromosomes. Epigenet Chromatin 8: 41. 10.1186/s13072-015-0033-5 PMC4589918 26430472

[emi15095-bib-0060] Selin, C. , de Kievit, T.R. , Belmonte, M.F. , and Fernando, W.G.D. (2016) Elucidating the role of effectors in plant‐fungal interactions: progress and challenges. Front Microbiol 7: 600. 27199930 10.3389/fmicb.2016.00600 PMC4846801

[emi15095-bib-0074] Shahi, S. , Beerens, B. , Bosch, M. , Linmans, J. , and Rep, M. (2016) Nuclear dynamics and genetic rearrangement in heterokaryotic colonies of Fusarium oxysporum. Fungal Genet Biol 91: 20–31. 27013267 10.1016/j.fgb.2016.03.003

[emi15095-bib-0061] Stergiopoulos, I. , and de Wit, P.J.G.M. (2009) Fungal effector proteins. Annu Rev Phytopathol 47: 233–263. 19400631 10.1146/annurev.phyto.112408.132637

[emi15095-bib-0062] Strom, N.B. , and Bushley, K.E. (2016) Two genomes are better than one: history, genetics, and biotechnological applications of fungal heterokaryons. Fung Biol Biotechnol 3: 4. 10.1186/s40694-016-0022-x PMC5611628 28955463

[emi15095-bib-0063] Sweigard, J.A. , Carroll, A.M. , Kang, S. , Farrall, L. , Chumley, F.G. , and Valent, B. (1995) Identification, cloning, and characterization of PWL2, a gene for host species specificity in the rice blast fungus. Plant Cell 7: 1221–1233. 7549480 10.1105/tpc.7.8.1221 PMC160946

[emi15095-bib-0064] Takken, F.L.W. , van Wijk, R. , Michielse, C.B. , Houterman, P.M. , Ram, A.F.J. , and Cornelissen, B.J.C. (2004) A one‐step method to convert vectors into binary vectors suited for agrobacterium‐mediated transformation. Curr Genet 45: 242–248. 14745506 10.1007/s00294-003-0481-5

[emi15095-bib-0065] Tsuge, T. , Harimoto, Y. , Akimitsu, K. , Ohtani, K. , Kodama, M. , Akagi, Y. , *et al* (2013) Host‐selective toxins produced by the plant pathogenic fungus *Alternaria alternata* . FEMS Microbiol Rev 37: 44–66. 22846083 10.1111/j.1574-6976.2012.00350.x

[emi15095-bib-0066] Vanheule, A. , Audenaert, K. , Warris, S. , van de Geest, H. , Schijlen, E. , Höfte, M. , *et al* (2016) Living apart together: crosstalk between the core and supernumerary genomes in a fungal plant pathogen. BMC Genomics 17: 670. 27552804 10.1186/s12864-016-2941-6 PMC4994206

[emi15095-bib-0067] Vlaardingerbroek, I. , Beerens, B. , Rose, L. , Fokkens, L. , Cornelissen, B.J.C. , and Rep, M. (2016a) Exchange of core chromosomes and horizontal transfer of lineage‐specific chromosomes in *Fusarium oxysporum* . Environ Microbiol 18: 3702–3713. 26941045 10.1111/1462-2920.13281

[emi15095-bib-0068] Vlaardingerbroek, I. , Beerens, B. , Schmidt, S.M. , Cornelissen, B.J.C. , and Rep, M. (2016b) Dispensable chromosomes in *Fusarium oxysporum* f. sp. lycopersici. Mol Plant Pathol 17: 1455–1466. 27271322 10.1111/mpp.12440 PMC6638487

[emi15095-bib-0069] Vlaardingerbroek, I. , Beerens, B. , Shahi, S. , and Rep, M. (2015) Fluorescence assisted selection of transformants (FAST): using flow cytometry to select fungal transformants. Fungal Genet Biol 76: 104–109. 25687933 10.1016/j.fgb.2015.02.003

[emi15095-bib-0070] Williams, A.H. , Sharma, M. , Thatcher, L.F. , Azam, S. , Hane, J.K. , Sperschneider, J. , *et al* (2016) Comparative genomics and prediction of conditionally dispensable sequences in legume‐infecting *Fusarium oxysporum* formae speciales facilitates identification of candidate effectors. BMC Genomics 17: 191. 26945779 10.1186/s12864-016-2486-8 PMC4779268

[emi15095-bib-0071] Van de Wouw, A.P. , Lowe, R.G.T. , Elliott, C.E. , Dubois, D.J. , and Howlett, B.J. (2014) An avirulence gene, AvrLmJ1, from the blackleg fungus, *Leptosphaeria maculans*, confers avirulence to *Brassica juncea* cultivars. Mol Plant Pathol 15: 523–530. 24279453 10.1111/mpp.12105 PMC6638781

[emi15095-bib-0072] Yoshida, K. , Saitoh, H. , Fujisawa, S. , Kanzaki, H. , Matsumura, H. , Yoshida, K. , *et al* (2009) Association genetics reveals three novel Avirulence genes from the rice blast fungal pathogen *Magnaporthe oryzae* . Plant Cell 21: 1573–1591. 19454732 10.1105/tpc.109.066324 PMC2700537

[emi15095-bib-0073] Zhang, S. , and Xu, J.‐R. (2014) Effectors and effector delivery in *Magnaporthe oryzae* . PLoS Pathog 10: e1003826. 24391496 10.1371/journal.ppat.1003826 PMC3879361

