# Supplementary material for: Partial pathogenicity chromosomes in Fusarium oxysporum are sufficient to cause disease and can be horizontally transferred
Source: Environ Microbiol. 2020 Jun 14;22(12):4985–5004. doi: 10.1111/1462-2920.15095 (PMC7818268; doi:10.1111/1462-2920.15095)
Supplement: Supplementary file 8 — Table S1. Summary of Agrobacterium‐mediated Fusarium transformations. [file EMI-22-4985-s008.docx]

**Table S1: Summary of *Agrobacterium*-mediated *Fusarium* transformation.**

| ATMT round | Plasmid used | Original strain | Number of transformants | Description |
| --- | --- | --- | --- | --- |
| ATMT_I | pRW1pPfem1RFP_SIX10/12/7 | 14HG6B | 396 | No in locus transformants; two transformants have lost *SIX10*, *SIX12* and close regions |
|  | pRW1pPfem1RFP_FOXG_16428 | 14HG6B | 406 | One in locus transformants, containing ectopic insertions |
| ATMT_II | pRW1pPfem1RFP_SIX10/12/7 | 14HG6B | 37 | No in locus transformant |
|  | pRW1pPfem1RFP_FOXG_14135 | 14HG6B | 60 | No in locus transformant |
|  | pRW1pPfem1RFP_FOXG_16428 | 14HG6B | 59 | No in locus transformant |
| ATMT_III | pRW1pPfem1RFP_FOXG_16428 | 14HG6B | 480 | No in locus transformant |
| ATMT_IV | pRW1pPfem1RFP_FOXG_14135 | 14HG6B | 240 | One in locus transformant, without ectopic insertions |
