# Supplementary material for: Partial pathogenicity chromosomes in Fusarium oxysporum are sufficient to cause disease and can be horizontally transferred
Source: Environ Microbiol. 2020 Jun 14;22(12):4985–5004. doi: 10.1111/1462-2920.15095 (PMC7818268; doi:10.1111/1462-2920.15095)
Supplement: Supplementary file 9 — Table S2. Fol pathogenicity chromosome deletion strains obtained. Symbols used in the table: + for positive PCR result, − for negative PCR result; grey regions without symbol for presumed presence, white regions without symbols for presumed absence. [file EMI-22-4985-s009.docx]

**Table S2: Fol pathogenicity chromosome deletion strains obtained.**

Symbols used in the table: + for positive PCR result, - for negative PCR result; grey regions without symbol for presumed presence, white regions without symbols for presumed absence.

| **Name** | ***c*** | ***g*** | ***GFP*** | ***SIX9*** | ***SIX6*** | ***ORX1*** | ***SIX11*** | ***Cen*** | ***SIX14*** | ***SIX2*** | ***SIX3*** | ***SIX5*** | ***SIX13*** | ***RFP*** | ***SIX10*** | ***SIX12*** | ***SIX7*** | ***SIX13*** |
| --- | --- | --- | --- | --- | --- | --- | --- | --- | --- | --- | --- | --- | --- | --- | --- | --- | --- | --- |
| **14HG6B** | **+** | **+** | **+** | **+** | **+** | **+** | **+** | **+** | **+** | **+** | **+** | **+** | **+** |  | **+** | **+** | **+** | **+** |
| **14HGPR** | **+** | **+** | **+** | **+** | **+** | **+** | **+** | **+** | **+** | **+** | **+** | **+** | **+** | **+** | **+** | **+** | **+** | **+** |
| **14-2** | **-** | **-** | **-** | **-** | **-** | **-** | **-** | **+** |  |  |  |  |  |  |  |  |  |  |
| **14-7** | **-** | **-** | **-** | **-** | **-** | **-** | **-** | **+** |  |  |  |  |  |  |  |  |  |  |
| **△GFP#1** | **-** | **-** | **-** | **-** | **+** |  |  |  |  |  |  |  |  | **+** |  |  |  |  |
| **△GFP#2** | **-** | **-** | **-** | **+** | **+** | **+** |  |  |  |  |  |  |  | **+** |  |  |  |  |
| **△GFP#3** | **-** | **-** | **-** | **+** | **+** |  |  |  |  |  |  |  |  | **+** |  |  |  |  |
| **△GFP#4** | **-** | **-** | **-** | **+** | **+** |  |  |  |  |  |  |  |  | **+** |  |  |  |  |
| **△GFP#5** | **-** | **-** | **-** | **+** | **+** |  |  |  |  |  |  |  |  | **+** |  |  |  |  |
| **△GFP#6** | **-** | **-** | **-** | **-** | **-** | **-** |  | **+** | **+** |  |  |  |  | **+** |  |  |  |  |
| **△GFP#7** | **-** | **-** | **-** | **-** | **+** | **+** |  |  |  |  |  |  |  | **+** |  |  |  |  |
| **△GFP#8** | **-** | **-** | **-** | **-** | **-** | **+** | **+** | **+** | **+** |  |  |  |  | **+** |  |  |  |  |
| **△GFP#9** | **+** | **-** | **-** | **-** | **+** |  |  |  |  |  |  |  |  | **+** |  |  |  |  |
| **△GFP#10** | **-** | **-** | **-** | **+** | **+** |  |  |  |  |  |  |  |  | **+** |  |  |  |  |
| **△GFP#11** | **-** | **-** | **-** | **+** | **+** | **+** |  |  |  |  |  |  |  | **+** |  |  |  |  |
| **△GFP#12** | **-** | **-** | **-** | **-** | **-** | **-** |  | **+** | **+** |  |  |  |  | **+** |  |  |  |  |
| **△GFP#13** | **-** | **-** | **-** | **-** | **-** | **-** |  | **+** | **+** |  |  |  |  | **+** |  |  |  |  |
| **△GFP#14** | **-** | **-** | **-** | **-** | **+** |  |  |  |  |  |  |  |  | **+** |  |  |  |  |
| **△GFP#15** | **-** | **-** | **-** | **+** | **+** |  |  |  |  |  |  |  |  | **+** |  |  |  |  |
| **△GFP#16** | **-** | **-** | **-** | **-** | **-** | **-** |  | **+** | **+** |  |  |  |  | **+** |  |  |  |  |
| **△GFP#17** | **-** | **-** | **-** | **-** | **+** |  |  |  |  |  |  |  |  | **+** |  |  |  |  |
| **△GFP#18** | **-** | **-** | **-** | **+** | **+** | **+** |  |  |  |  |  |  |  | **+** |  |  |  |  |
| **△GFP#19** | **+** | **+** | **-** | **+** | **+** | **+** | **+** | **+** | **+** | **+** |  | **+** |  | **+** |  |  |  |  |
| **△GFP#20** | **-** | **-** | **-** | **-** | **-** | **-** | **-** | **-** | **+** |  | **+** |  |  | **+** | **+** | **+** | **+** |  |
| **△GFP#21** | **+** | **-** | **-** | **+** | **+** | **+** | **+** | **+** | **+** | **+** |  | **+** |  | **+** |  |  |  |  |
| **△GFP#22** | **-** | **-** | **-** | **-** | **-** | **-** | **-** | **+** | **+** | **+** | **+** | **+** | **+** | **+** |  |  |  |  |
| **△GFP#23** | **-** | **-** | **-** | **+** | **+** | **+** | **+** | **+** | **+** | **+** | **+** | **+** | **+** | **+** |  |  |  |  |
| **△GFP#24** | **+** | **+** | **-** | **+** | **+** | **+** | **+** | **+** |  |  |  |  |  | **+** |  |  |  |  |
| **△GFP#25** | **+** | **+** | **-** | **+** | **+** | **+** | **+** | **+** |  |  |  |  |  | **+** |  |  |  |  |
| **△GFP#26** | **-** | **-** | **-** | **-** | **-** | **-** | **-** | **+** | **+** | **+** |  | **+** |  | **+** |  |  |  |  |
| **△GFP#27** | **-** | **-** | **-** | **-** | **+** | **-** | **+** | **+** | **+** | **+** |  | **+** |  | **+** |  |  |  |  |
| **△GFP#28** | **-** | **-** | **-** | **-** | **+** | **-** | **+** | **+** |  |  |  |  |  | **+** |  |  |  |  |
| **△GFP#29** | **-** | **-** | **-** | **+** | **+** | **+** | **+** | **+** | **+** | **+** |  | **+** |  | **+** |  |  |  |  |
| **△GFP#30** | **-** | **-** | **-** | **-** | **-** | **-** | **+** | **-** | **+** |  | **+** | **+** |  | **+** | **+** | **+** | **+** |  |
| **△GFP#31** | **+** | **+** | **-** | **+** | **+** | **+** | **+** | **+** |  |  |  |  |  | **+** |  |  |  |  |
| **△GFP#32** | **-** | **-** | **-** | **+** | **+** | **+** | **+** | **+** |  |  |  |  |  | **+** |  |  |  |  |
| **△GFP#33** | **+** | **+** | **-** | **+** | **+** | **+** | **+** | **+** |  |  |  |  |  | **+** |  |  |  |  |
| **△GFP#34** | **+** | **-** | **-** | **-** | **+** | **+** | **+** | **+** | **+** | **+** | **+** | **+** | **+** | **+** |  |  |  |  |
| **△GFP#35** | **-** | **-** | **-** | **+** | **+** | **+** | **+** | **+** |  |  |  |  |  | **+** |  |  |  |  |
| **△GFP#36** | **-** | **-** | **-** | **+** | **+** | **+** | **+** | **+** | **+** |  |  |  |  | **+** | **+** | **+** | **+** |  |
| **△GFP#37** | **+** | **+** | **-** | **+** | **+** | **+** | **+** | **+** |  |  |  |  |  | **+** |  |  |  |  |
| **△GFP#38** | **+** | **-** | **-** | **-** | **+** | **+** | **+** | **+** | **+** | **+** |  | **+** |  | **+** |  |  |  |  |
| **△GFP#39** | **+** | **+** | **-** | **+** | **+** | **+** | **+** | **+** |  |  |  |  |  | **+** |  |  |  |  |
| **△GFP#40** | **-** | **-** | **-** | **-** | **-** | **-** | **-** | **+** | **+** | **+** | **+** | **+** | **+** | **+** |  |  |  |  |
| **△GFP#41** | **-** | **-** | **-** | **-** | **+** | **+** | **+** | **+** | **+** | **+** |  | **+** |  | **+** |  |  |  |  |
| **△GFP#42** | **+** | **+** | **-** | **+** | **+** | **+** | **+** | **+** |  |  |  |  |  | **+** |  |  |  |  |
| **△GFP#43** | **+** | **+** | **-** | **+** | **+** | **+** | **+** | **+** |  |  |  |  |  | **+** |  |  |  |  |
| **△RFP#1** |  |  | **+** |  |  |  |  |  | **+** | **+** |  | **+** |  | **-** |  |  | **+** |  |
| **△RFP#2** |  |  | **+** |  |  |  |  |  | **+** | **+** |  | **+** |  | **-** |  |  | **+** |  |
| **△RFP#3** |  |  | **+** |  |  |  |  |  | **+** | **+** |  | **+** |  | **-** |  |  | **+** |  |
| **△RFP#4** |  |  | **+** |  |  |  |  |  | **+** | **+** |  | **+** |  | **-** |  |  | **+** |  |
| **△RFP#5** |  |  | **+** |  |  |  |  |  | **+** | **+** |  | **+** |  | **-** |  |  | **+** |  |
| **△RFP#6** |  |  | **+** |  |  |  |  |  | **+** | **+** |  | **+** |  | **-** |  |  | **+** |  |
| **△RFP#7** |  |  | **+** |  |  |  |  |  | **+** | **+** |  | **+** |  | **-** |  |  | **+** |  |
| **△RFP#8** |  |  | **+** |  |  |  |  |  | **+** | **+** |  | **+** |  | **-** |  |  | **+** |  |
| **△RFP#9** |  |  | **+** |  |  |  |  |  | **+** | **+** |  | **+** |  | **-** |  |  | **+** |  |
| **△RFP#10** |  |  | **+** |  |  |  |  |  | **+** | **+** |  | **+** |  | **-** |  |  | **+** |  |
| **△RFP#11** | **+** | **+** | **+** | **+** | **+** | **+** | **+** | **+** | **+** | **+** |  | **+** | **+** | **-** | **-** | **-** | **-** |  |
| **△RFP#12** | **+** | **+** | **+** | **+** | **+** | **+** | **+** | **+** | **-** | **-** |  | **-** | **-** | **-** | **-** | **-** | **-** | **-** |
| **△RFP#13** |  |  | **+** |  |  |  |  |  | **+** | **+** |  | **+** |  | **-** |  |  | **+** |  |
| **△RFP#14** |  |  | **+** | **+** |  |  | **+** | **+** | **+** | **+** | **-** | **-** | **-** | **-** |  |  | **-** | **-** |
| **△RFP#15** |  |  | **+** |  |  |  |  | **+** | **+** | **+** | **+** | **+** |  | **-** |  |  | **+** |  |
| **△RFP#16** |  |  | **+** | **+** |  |  | **+** | **-** | **-** | **-** | **-** | **-** | **-** | **-** |  |  | **-** | **-** |
| **△RFP#17** |  |  | **+** |  |  |  |  | **+** | **+** | **+** | **+** | **+** |  | **-** |  |  | **+** |  |
| **△RFP#18** |  |  | **+** |  |  |  |  | **+** | **+** | **+** | **+** | **+** |  | **-** |  |  | **+** |  |
