# Supplementary material for: Partial pathogenicity chromosomes in Fusarium oxysporum are sufficient to cause disease and can be horizontally transferred
Source: Environ Microbiol. 2020 Jun 14;22(12):4985–5004. doi: 10.1111/1462-2920.15095 (PMC7818268; doi:10.1111/1462-2920.15095)
Supplement: Supplementary file 10 — Table S3. Summary of three fluorescence assisted cell sorting (FACS) experiments. [file EMI-22-4985-s010.docx]

**Table S3. Summary of three Fluorescence Assisted Cell Sorting (FACS) experiments.**

| **Culture** | **FACS_II (5 cultures)** | | **FACS_III (5 cultures)** | | **FACS_IV (10 cultures)** | |
| --- | --- | --- | --- | --- | --- | --- |
| **FACS run** | Loss of red fluorescence | Loss of green fluorescence | Loss of red fluorescence | Loss of green fluorescence | Loss of red fluorescence | Loss of green fluorescence |
| **Total spores** | 8983381 | 6098306 | 5218645 | 2129312 | 21200000 | - |
| **Deflected spores** | 226 | 250 | 375 | 250 | 289 | - |
| **Colonies formed on PDA plates** | 199 | 27 | 335 | 74 | 246 | - |
| **Loss of fluorescence confirmed microscopically** | 3 | 20 | 10 | 63 | 5 | - |
| **Loss of fluorescence confirmed by PCR** | 3 | 18 | 10 | 63 | 5 | - |
| **Minimal number of different chromosomal deletions** | 3 | 12 | 6 | 45 | 5 | - |
| **RFP or GFP gene loss strains / 4 million spores** | 1,3 | 13 | 4,6 | 184 | 1,0 | - |
