# Supplementary material for: Partial pathogenicity chromosomes in Fusarium oxysporum are sufficient to cause disease and can be horizontally transferred
Source: Environ Microbiol. 2020 Jun 14;22(12):4985–5004. doi: 10.1111/1462-2920.15095 (PMC7818268; doi:10.1111/1462-2920.15095)
Supplement: Supplementary file 11 — Table S4. Details of the second fluorescence assisted cell sorting (FACS) experiment. [file EMI-22-4985-s011.docx]

**Table S4. Details of the second Fluorescence Assisted Cell Sorting (FACS) experiment.**

| **Culture** | **FACS_II_14HGPR-1** | | **FACS_II_14HGPR-2** | | **FACS_II_14HGPR-3** | | **FACS_II_14HGPR-4** | | **FACS_II_14HGPR-5** | |
| --- | --- | --- | --- | --- | --- | --- | --- | --- | --- | --- |
| **FACS run** | 14HGPR-1△GFP | 14HGPR-1△RFP | 14HGPR-2△GFP | 14HGPR-2△RFP | 14HGPR-3△GFP | 14HGPR-3△RFP | 14HGPR-4△GFP | 14HGPR-4△RFP | 14HGPR-5△GFP | 14HGPR-5△RFP |
| **Total spores** | 1363451 | 1983381 | 1258425 | 2000000 | 1839232 | 2000000 | 259534 | 2000000 | 1377664 | 1000000 |
| **Deflected spores** | 50 | 150 | 50 | 25 | 50 | 50 | 50 | 0 | 50 | 1 |
| **Colonies formed on PDA plates** | 1 | 131 | 13 | 22 | 8 | 46 | 1 | - | 4 | 0 |
| **Loss of fluorescence confirmed by microscopy** | 1 | 2 | 12 | 1 | 5 | 1 | 0 | - | 3 | - |
| **RFP or GFP gene loss strains** | 1 | 2 | 11 | 1 | 5 | 0 | - | - | 3 | - |
| **RFP or GFP gene loss strains / 4 million spores** | 3 | 4 | 35 | 2 | 11 | - | - | - | 9 | - |
