# Supplementary material for: Partial pathogenicity chromosomes in Fusarium oxysporum are sufficient to cause disease and can be horizontally transferred
Source: Environ Microbiol. 2020 Jun 14;22(12):4985–5004. doi: 10.1111/1462-2920.15095 (PMC7818268; doi:10.1111/1462-2920.15095)
Supplement: Supplementary file 12 — Table S5. Details of the third fluorescence assisted cell sorting (FACS) experiment. [file EMI-22-4985-s012.docx]

**Table S5. Details of the third Fluorescence Assisted Cell Sorting (FACS) experiment.**

| **Culture** | **FACS_III_14HGPR-1** | | **FACS_III_14HGPR-2** | | **FACS_III_14HGPR-3** | | **FACS_III_14HGPR-4** | | **FACS_III_14HGPR-5** | |
| --- | --- | --- | --- | --- | --- | --- | --- | --- | --- | --- |
| **FACS run** | 14HGPR-1△GFP | 14HGPR-1△RFP | 14HGPR-2△GFP | 14HGPR-2△RFP | 14HGPR-3△GFP | 14HGPR-3△RFP | 14HGPR-4△GFP | 14HGPR-4△RFP | 14HGPR-5△GFP | 14HGPR-5△RFP |
| **Total spores** | 619883 | 959552 | 337800 | 1485529 | 215871 | 958565 | 345356 | 1518441 | 610402 | 296558 |
| **Deflected spores** | 50 | 75 | 50 | 75 | 50 | 75 | 50 | 75 | 50 | 75 |
| **Colonies formed on PDA plates** | 28 | 69 | 7 | 66 | 8 | 62 | 6 | 64 | 25 | 74 |
| **Loss of fluorescence confirmed by microscopy** | 25 | 0 | 5 | 2 | 6 | 8 | 5 | 5 | 22 | 2 |
| **RFP or GFP gene loss strains** | 25 | 0 | 5 | 1 | 6 | 1 | 5 | 5 | 22 | 2 |
| **RFP or GFP gene loss strains / 4 million spores** | 161 | - | 59 | 2,7 | 111 | 4,0 | 58 | 13 | 144 | 27 |
