# Supplementary material for: Partial pathogenicity chromosomes in Fusarium oxysporum are sufficient to cause disease and can be horizontally transferred
Source: Environ Microbiol. 2020 Jun 14;22(12):4985–5004. doi: 10.1111/1462-2920.15095 (PMC7818268; doi:10.1111/1462-2920.15095)
Supplement: Supplementary file 13 — Table S6. Details of the fourth fluorescence assisted cell sorting (FACS) experiment. [file EMI-22-4985-s013.docx]

**Table S6. Details of the fourth Fluorescence Assisted Cell Sorting (FACS) experiment.**

| **Culture** | **FACS_IV_14HGPR-1** | **FACS_IV_14HGPR-2** | **FACS_IV_14HGPR-3** | **FACS_IV_14HGPR-4** | **FACS_IV_14HGPR-5** | **FACS_IV_14HGPR-6** | **FACS_IV_14HGPR-7** | **FACS_IV_14HGPR-8** | **FACS_IV_14HGPR-9** | **FACS_IV_14HGPR-10** |
| --- | --- | --- | --- | --- | --- | --- | --- | --- | --- | --- |
| **FACS run** | **△RFP** | **△RFP** | **△RFP** | **△RFP** | **△RFP** | **△RFP** | **△RFP** | **△RFP** | **△RFP** | **△RFP** |
| **Total spores** | 200000 | 1000000 | 1000000 | 2000000 | 1000000 | 5000000 | 6000000 | 3000000 | 1000000 | 1000000 |
| **Deflected spores** | 50 | 2 | 4 | 50 | 7 | 50 | 50 | 50 | 21 | 5 |
| **Colonies formed on PDA plates** | 42 | 1 | 3 | 42 | 5 | 42 | 41 | 42 | 15 | 3 |
| **Loss of fluorescence confirmed by microscopy** | 1 | 0 | 0 | 0 | 0 | 0 | 2 | 1 | 1 | 0 |
| **RFP or GFP gene loss strains** | 1 | - | - | - | - | - | 2 | 1 | 1 | - |
| **RFP or GFP gene loss strains / 4 million spores** | 20 | - | - | - | - | - | 1,3 | 1,3 | 4,0 | - |
