# Supplementary material for: Partial pathogenicity chromosomes in Fusarium oxysporum are sufficient to cause disease and can be horizontally transferred
Source: Environ Microbiol. 2020 Jun 14;22(12):4985–5004. doi: 10.1111/1462-2920.15095 (PMC7818268; doi:10.1111/1462-2920.15095)
Supplement: Supplementary file 14 — Table S7. Strains and single chromosomes sent for sequencing. [file EMI-22-4985-s014.docx]

**Table S7: Strains and single chromosomes sent for sequencing.**

| **Name** | **Description** | **Sequencing platform** |
| --- | --- | --- |
| Fol4248 | Wild type | Illumina (Broad) |
| Fol4248 | Wild type | Pacbio |
| △GFP#8 | Loss of GFP deletion strain | Illumina |
| △GFP#20 | Loss of GFP deletion strain | Illumina |
| △GFP#22 | Loss of GFP deletion strain | Illumina |
| △GFP#26 | Loss of GFP deletion strain | Illumina |
| △GFP#27 | Loss of GFP deletion strain | Illumina |
| △GFP#29 | Loss of GFP deletion strain | Illumina |
| △RFP#11 | Loss or RFP deletion strain | Illumina |
| △RFP#12 | Loss or RFP deletion strain | Illumina |
| △RFP#14 | Loss or RFP deletion strain | Illumina |
| △RFP#16 | Loss or RFP deletion strain | Illumina |
| HCT_△GFP#29-2 | HCT Strain | Illumina |
| HCT_△GFP#8-2 | HCT Strain | Illumina |
| HCT_△GFP#26-1 | HCT Strain | Illumina |
| △GFP#8_SC | Single chromosome | Illumina |
| HCT_△GFP#8_SC | Single chromosome | Illumina |
| △GFP#26_SC | Single chromosome | Illumina |
| HCT_△GFP#26_SC | Single chromosome | Illumina |
| △RFP#11_SC | Single chromosome | Illumina |
| △RFP#12_SC | Single chromosome | Illumina |
| △GFP#29_SC_L | Single chromosome | Illumina |
| △GFP#29_SC_S | Single chromosome | Illumina |
| △GFP#29_SC_XS | Single chromosome | Illumina |
| HCT_△GFP#29_SC_L | Single chromosome | Illumina |
| HCT_△GFP#29_SC_S | Single chromosome | Illumina |
