# Supplementary material for: Partial pathogenicity chromosomes in Fusarium oxysporum are sufficient to cause disease and can be horizontally transferred
Source: Environ Microbiol. 2020 Jun 14;22(12):4985–5004. doi: 10.1111/1462-2920.15095 (PMC7818268; doi:10.1111/1462-2920.15095)
Supplement: Supplementary file 15 — Table S8. Summary of Horizontal Chromosome Transfer (HCT) experiments. No: no successful transfer; Yes: successful transfer. Only strains for which transfer was attempted are shown in this table. [file EMI-22-4985-s015.docx]

**Table S8. Summary of Horizontal Chromosome Transfer (HCT).**

No: no successful transfer; Yes: successful transfer. Only strains for which transfer was attempted are shown in this table.

|  | ***c*** | ***g*** | ***GFP*** | ***SIX9*** | ***SIX6*** | ***ORX1*** | ***SIX11*** | ***Cen*** | ***SIX14*** | ***SIX2*** | ***SIX3*** | ***SIX5*** | ***SIX13*** | ***RFP*** | ***SIX10*** | ***SIX12*** | ***SIX7*** | ***SIX13*** | **HCT_I** | **HCT_II** | **HCT_III** | **HCT_IV** | **HCT_V** |
| --- | --- | --- | --- | --- | --- | --- | --- | --- | --- | --- | --- | --- | --- | --- | --- | --- | --- | --- | --- | --- | --- | --- | --- |
| △GFP#2 | - | - | - | + | + | + |  |  |  |  |  |  |  | + |  |  |  |  | No |  |  |  |  |
| △GFP#6 | - | - | - | - | - | - |  | + | + |  |  |  |  | + |  |  |  |  | No |  |  | No | No |
| **△GFP#8** | - | - | - | - | - | + | + | + | + |  |  |  |  | + |  |  |  |  |  |  | Yes |  |  |
| △GFP#12 | - | - | - | - | - | - |  | + | + |  |  |  |  | + |  |  |  |  | No |  |  | No | No |
| △GFP#18 | - | - | - | + | + | + |  |  |  |  |  |  |  | + |  |  |  |  | No |  |  |  |  |
| △GFP#19 | + | + | - | + | + | + | + | + | + | + |  | + |  | + |  |  |  |  |  |  |  |  | No |
| △GFP#20 | - | - | - | - | - | - | - | - | + |  | + |  |  | + | + | + | + |  |  |  |  |  | No |
| △GFP#22 | - | - | - | - | - | - | - | + | + | + | + | + | + | + |  |  |  |  |  | No |  |  | No |
| △GFP#23 | - | - | - | + | + | + | + | + | + | + | + | + | + | + |  |  |  |  |  | No |  |  |  |
| △GFP#24 | + | + | - | + | + | + | + | + |  |  |  |  |  | + |  |  |  |  |  |  | No |  |  |
| **△GFP#26** | - | - | - | - | - | - | - | + | + | + |  | + |  | + |  |  |  |  |  | No |  | No | Yes |
| △GFP#27 | - | - | - | - | + | - | + | + | + | + |  | + |  | + |  |  |  |  |  |  | No |  |  |
| **△GFP#29** | - | - | - | + | + | + | + | + | + | + |  | + |  | + |  |  |  |  |  | Yes |  |  |  |
| △GFP#37 | + | + | - | + | + | + | + | + |  |  |  |  |  | + |  |  |  |  |  |  |  |  | No |
| △GFP#40 | - | - | - | - | - | - | - | + | + | + | + | + | + | + |  |  |  |  |  | No |  |  | No |
| △GFP#41 | - | - | - | - | + | + | + | + | + | + |  | + |  | + |  |  |  |  |  |  | No |  |  |
| **△RFP#1** |  |  | + |  |  |  |  |  | + | + |  | + |  | - |  |  | + |  |  |  |  |  | Yes |
| △RFP#2 |  |  | + |  |  |  |  |  | + | + |  | + |  | - |  |  | + |  |  |  |  |  | No |
| △RFP#7 |  |  | + |  |  |  |  |  | + | + |  | + |  | - |  |  | + |  |  |  |  |  | No |
| △RFP#11 | + | + | + | + | + | + | + | + | + | + |  | + | + | - | - | - | - | - |  | No | No | No | No |
| △RFP#12 | + | + | + | + | + | + | + | + | - | - |  | - | - | - | - | - | - | - |  | No | No | No | No |
| △RFP#14 |  |  | + | + |  |  | + | + | + | + | - | - | - | - |  |  | - | - |  |  | No | No | No |
| △RFP#16 |  |  | + | + |  |  | + | - | - | - | - | - | - | - |  |  | - | - |  |  |  |  | No |
| △RFP#18 |  |  | + |  |  |  |  | + | + | + | + | + |  | - |  |  | + |  |  |  | No |  |  |
