# Supplementary material for: Partial pathogenicity chromosomes in Fusarium oxysporum are sufficient to cause disease and can be horizontally transferred
Source: Environ Microbiol. 2020 Jun 14;22(12):4985–5004. doi: 10.1111/1462-2920.15095 (PMC7818268; doi:10.1111/1462-2920.15095)
Supplement: Supplementary file 16 — Table S9. Different strain ratios and media used in five HCT experiments. PDA: potato dextrose agar; CDA: Czapek Dox Agar. CAT medium: 0.17% YNB, 25 mM KNO3. [file EMI-22-4985-s016.docx]

**Table S9. Different strain ratios and medium used in five HCT experiments.**

PDA: potato dextrose agar; CDA: Czapek Dox Agar. CAT medium: 0.17% YNB, 25mM KNO_3._

|  | **HCT_I** | **HCT_II** | **HCT_III** | **HCT_IV** | **HCT_V** |
| --- | --- | --- | --- | --- | --- |
| **Number of donor strains** | 4 | 7 | 8 | 6 | 15 |
| **CAT medium pre-incubation** | no | no | no | yes | no |
| **Donor:recipient ratio** | 1:1 | 1:1; 5:1; 10:1 | 1:1; 10:1; 20:1 | 10:1 | 1:1 |
| **Co-incubation medium** | PDA | PDA | PDA; CDA | PDA | PDA; CDA |
