# Supplementary material for: Partial pathogenicity chromosomes in Fusarium oxysporum are sufficient to cause disease and can be horizontally transferred
Source: Environ Microbiol. 2020 Jun 14;22(12):4985–5004. doi: 10.1111/1462-2920.15095 (PMC7818268; doi:10.1111/1462-2920.15095)
Supplement: Supplementary file 17 — Table S10. Horizontal chromosome transfer was confirmed by PCR. Symbols used in the table: + for positive PCR result, − for negative PCR result; black regions without symbol are presumed to be present, white regions without symbols are presumed to be absent. [file EMI-22-4985-s017.docx]

**Table S10. Horizontal chromosome transfer was confirmed by PCR.**

Symbols used in the table: + for positive PCR result, - for negative PCR result; black regions without symbol are presumed to be present, white regions without symbols are presumed to be absent.

|  | ***SCAR*** | ***HYG2*** | ***BLE2*** | ***c*** | ***g*** | ***HYG1*** | ***GFP*** | ***SIX9*** | ***SIX6*** | ***SIX11*** | ***ORX1*** | ***CenL*** | ***Cen*** | ***SIX14*** | ***SIX2*** | ***SIX3*** | ***SIX5*** | ***SIX13*** | ***BLE1*** | ***RFP*** | ***SIX10*** | ***SIX12*** | ***SIX7*** | ***SIX13*** |
| --- | --- | --- | --- | --- | --- | --- | --- | --- | --- | --- | --- | --- | --- | --- | --- | --- | --- | --- | --- | --- | --- | --- | --- | --- |
| 14HGPR |  |  |  | + | + | + | + | + | + | + | + |  | + | + | + | + | + | + | + | + | + | + | + | + |
| △GFP#8 | - |  |  | - | - | - | - | - | - | + | + |  | + | + |  |  | + |  | + | + |  |  |  |  |
| HCT_△GFP#8-1 | + | + |  |  |  |  |  | - | - | + | + |  | + |  |  |  | + |  | + |  |  |  |  |  |
| HCT_△GFP#8-2 | + | + |  |  |  |  |  | - | - | + | + |  | + |  |  |  | + |  | + |  |  |  |  |  |
| HCT_△GFP#8-3 | + | + |  |  |  |  |  | - | - | + | + |  | + |  |  |  | + |  | + |  |  |  |  |  |
| HCT_△GFP#8-4 | + | + |  |  |  |  |  | - | - | + | + |  | + |  |  |  | + |  | + |  |  |  |  |  |
| HCT_△GFP#8-5 | + | + |  |  |  |  |  | - | - | + | + |  | + |  |  |  | + |  | + |  |  |  |  |  |
| HCT_△GFP#8-6 | + | + |  |  |  |  |  | - | - | + | + |  | + |  |  |  | + |  | + |  |  |  |  |  |
| HCT_△GFP#8-7 | + | + |  |  |  |  |  | - | - |  | + |  | + |  |  |  | + |  | + |  |  |  |  |  |
| HCT_△GFP#8-8 | + | + |  |  |  |  |  | - | - | + | + |  | + |  |  |  | + |  | + |  |  |  |  |  |
| HCT_△GFP#8-9 | + | + |  |  |  |  |  | - | - | + | + |  | + |  |  |  | + |  | + |  |  |  |  |  |
| HCT_△GFP#8-10 | + | + |  |  |  |  |  | - | - | + | + |  | + |  |  |  | + |  | + |  |  |  |  |  |
| △GFP#26 | - |  |  | - |  | - | - | - | - | - | - | - | + | + | + |  | + |  | + | + |  |  |  |  |
| HCT_△GFP#26-1 | + | + |  |  |  |  |  | - |  | - | - | - | + |  |  |  | + |  | + |  |  |  |  |  |
| HCT_△GFP#26-2 | + | + |  |  |  |  |  | - |  | - | - | - | + |  |  |  | + |  | + |  |  |  |  |  |
| HCT_△GFP#26-3 | + | + |  |  |  |  |  | - |  | - | - | - | + |  |  |  | + |  | + |  |  |  |  |  |
| HCT_△GFP#26-4 | + | + |  |  |  |  |  | - |  | - | - | - | + |  |  |  | + |  | + |  |  |  |  |  |
| HCT_△GFP#26-5 | + | + |  |  |  |  |  | - |  | - | - | - | + |  |  |  | + |  | + |  |  |  |  |  |
| HCT_△GFP#26-6 | + | + |  |  |  |  |  | - |  | - | - | - | + |  |  |  | + |  | + |  |  |  |  |  |
| HCT_△GFP#26-7 | + | + |  |  |  |  |  | - |  | - | - | - | + |  |  |  | + |  | + |  |  |  |  |  |
| △GFP#29 | - |  |  | - | - | - | - | + | + | + | + |  | + | + | + |  | + |  | + | + |  |  |  |  |
| HCT_△GFP#29-1 | + | + |  |  |  |  |  | + |  | + |  |  | + |  |  |  | + |  | + |  |  |  |  |  |
| HCT_△GFP#29-2 | + | + |  |  |  |  |  | + |  | + |  |  | + |  |  |  | + |  | + |  |  |  |  |  |
| HCT_△GFP#29-3 | + | + |  |  |  |  |  | + |  | + |  |  | + |  |  |  | + |  | + |  |  |  |  |  |
| HCT_△GFP#29-4 | + | + |  |  |  |  |  | + |  | + |  |  | + |  |  |  | + |  | + |  |  |  |  |  |
| HCT_△GFP#29-5 | + | + |  |  |  |  |  | + |  | + |  |  | + |  |  |  | + |  | + |  |  |  |  |  |
| HCT_△GFP#29-6 | + | + |  |  |  |  |  | + |  | + |  |  | + |  |  |  | + |  | + |  |  |  |  |  |
| △RFP#1 | - |  |  |  |  | + | + |  |  |  |  |  |  | + | + |  | + |  |  | - |  |  | + |  |
| HCT_△RFP#1-1 | + |  | + |  |  | + |  | + |  | + |  |  | + |  |  |  | + |  |  | - |  |  |  |  |
| HCT_△RFP#1-2 | + |  | + |  |  | + |  | + |  | + |  | + |  |  |  |  | + |  |  | - |  |  |  |  |
| HCT_△RFP#1-3 | + |  | + |  |  | + |  | + |  | + |  |  | + |  |  |  | + |  |  | - |  |  |  |  |
| HCT_△RFP#1-4 | + |  | + |  |  | + |  | + |  | + |  |  | + |  |  |  | + |  |  | - |  |  |  |  |
| HCT_△RFP#1-5 | + |  | + |  |  | + |  | + |  | + |  |  | + |  |  |  | + |  |  | - |  |  |  |  |
| HCT_△RFP#1-6 | + |  | + |  |  | + |  | + |  | + |  |  | + |  |  |  | + |  |  | - |  |  |  |  |
| HCT_△RFP#1-7 | + |  | + |  |  | + |  | + |  | + |  |  | + |  |  |  | + |  |  | - |  |  |  |  |
| Fo47_hgy1 | + | + | - |  |  |  | + | - | - | - | - | - | - |  |  |  | - |  |  |  |  |  |  |  |
| Fo47_GRB1 | + | - | + |  |  |  |  |  |  |  |  |  |  |  |  |  |  |  |  |  |  |  |  |  |
