# Supplementary material for: Partial pathogenicity chromosomes in Fusarium oxysporum are sufficient to cause disease and can be horizontally transferred
Source: Environ Microbiol. 2020 Jun 14;22(12):4985–5004. doi: 10.1111/1462-2920.15095 (PMC7818268; doi:10.1111/1462-2920.15095)
Supplement: Supplementary file 18 — Table S11. Primers used for cloning. [file EMI-22-4985-s018.docx]

**Table S11: Primers used for cloning.**

| FP number | Name | Sequence | Size of PCR product (bp) | Target sequence |
| --- | --- | --- | --- | --- |
| FP6992 | RFP-XbaI-F | AAAtctagaATGGCCTCCTCCGAGGACG | 684 | RFP |
| FP6993 | RFP-BglII-R | TTTagatctTTAGGCGCCGGTGGAGTGG |  |  |
| FP7075 | HiFi-six10/12/7-LF-F | cgttgtaaaacgacggccagtgccaCGTTGATTGATTAGCAAGC | 1117 | Upstream of SIX10 |
| FP7076 | HiFi-six10/12/7-LF-R | tatgccctttgtggcttatactccaCAATAGTCACTGGCTGCAG |  |  |
| FP7077 | HiFi-six10/12/7-RF-F | aggatccccgggtaccgagctcgatGAGTGCTATACCTATGCTCG | 892 | Downstream of SIX7 |
| FP7078 | HiFi-six10/12/7-RF-R | acatgattacgaattcttataagatCAACTAATCGCTACTAGCAGTC |  |  |
| FP7172 | HiFi-FOXG_14135-LF-F | cgttgtaaaacgacggccagtgccaAGAAAGGGGTTCTGAAGG | 1052 | Upstream of FOXG_14135 |
| FP7173 | HiFi-FOXG_14135-LF-R | tatgccctttgtggcttatactccaGTAGGAAACTTCGATGACTACC |  |  |
| FP7174 | HiFi-FOXG_14135-RF-F | aggatccccgggtaccgagctcgatACCCAAGACAGAAGGCGA | 941 | Downstream of FOXG_14135 |
| FP7175 | HiFi-FOXG_14135-RF-R | acatgattacgaattcttataagatCCGTAGACTCTTTCCTACCC |  |  |
| FP7176 | HiFi-FOXG_16428-LF-F | cgttgtaaaacgacggccagtgccaATCGAAGATCCAACGAAC | 1078 | Upstream of FOXG_16428 |
| FP7177 | HiFi-FOXG_16428-LF-R | tatgccctttgtggcttatactccaCAATGTGCAATGTTAGAACG |  |  |
| FP7178 | HiFi-FOXG_16428-RF-F | aggatccccgggtaccgagctcgatTCGACCTACGCCAGTCGTT | 975 | Downstream of FOXG_16428 |
| FP7179 | HiFi-FOXG_16428-RF-R | acatgattacgaattcttataagatCGATCTCATGCTCGGCAG |  |  |
