# Supplementary material for: Partial pathogenicity chromosomes in Fusarium oxysporum are sufficient to cause disease and can be horizontally transferred
Source: Environ Microbiol. 2020 Jun 14;22(12):4985–5004. doi: 10.1111/1462-2920.15095 (PMC7818268; doi:10.1111/1462-2920.15095)
Supplement: Supplementary file 19 — Table S12. Markers on the pathogenicity chromosome. [file EMI-22-4985-s019.docx]

**Table S12: Markers on the pathogenicity chromosome.**

| **FP number** | **Name** | **Sequence** | **Size of PCR product (bp)** | **Target sequence** |
| --- | --- | --- | --- | --- |
| FP4954 | FOXG_14164F | TACAGCCAGCTCAATAAGG | 518 | FOXG_14164 (c) |
| FP4955 | FOXG_14164R | TTATGTGTGTCGCTAGGTTC |  |  |
| FP4852 | FOXG_14188F2 | TTACTAACGTGATTGAACGG | 527 | FOXG_14188 (g) |
| FP4853 | FOXG_14188R2 | AACATGAACAGGCTCCATCC |  |  |
| FP732 | GFP-F-Apa1 | AAAGGGCCCATGGTGAGCAAGGGCGAGGAG | 732 | GFP |
| FP733 | GFP-R-Apa1 | AAAGGGCCCTTACTTGTACAGCTCGTCC |  |  |
| FP4455 | SIX9-F2 | CTTCTAGCAGTTGTAGCCAC | 323 | SIX9 |
| FP4456 | SIX9-R2 | GTACGCCAGTTGACGCAAG |  |  |
| FP1490 | SIX6-F1 | CTCTCCTGAACCATCAACTT | 793 | SIX6 |
| FP1491 | SIX6-R1 | CAAGACCAGGTGTAGGCATT |  |  |
| FP2014 | ORX1-F1 | GTTTGATCAGCCAGTTGTC | 2294 | ORX1 |
| FP2015 | ORX1-R1 | ATTCCGGGCCATTTGGGTTC |  |  |
| FP7895 | SIX11_F | TCAGATGCAGGGTCTATTGAG | 333 | SIX11 |
| FP7896 | SIX11_R | ATGATGTTCTCCAAAGCCATCC |  |  |
| FP8115 | Cen_L_F | GACTCGAACGACTTGATGACG | 966 | Centromere_L |
| FP8116 | Cen_L_R | GTGCACTATGTATAGAACAGC |  |  |
| FP2545 | PRED.PRO.16.F1 | AGTTACCAGCCAAAGTGATCG | 1359 | Centromere_R |
| FP24546 | PRED.PRO.16.R1 | CAGCACTTAGACCTTGCCTTG |  |  |
| FP7888 | SIX14F | CCACTATCTTGCCACCTATGC | 336 | SIX14 |
| FP7889 | SIX14R | CCTTAACAGCTGGTGGCTAG |  |  |
| FP998 | SIX2-F1 | ATGCTCTTCAAAATCGCGTG | 584 | SIX2 |
| FP999 | SIX2-R1 | TCAACATAGGCCACACCATT |  |  |
| FP2848 | six3realtime1 | ACTGATTGTGGCTGGACCTC | 532 | SIX3 |
| FP1364 | 2D4-F13 | ATCTGCTTGCACCCCAGCC |  |  |
| FP1488 | SIX5-F1 | ACACGCTCTACTACTCTTCA | 667 | SIX5 |
| FP1489 | SIX5-R1 | GAAAACCTCAACGCGGCAAA |  |  |
| FP6681 | ForcSIX13_ORFf | CTCCTGGTTCTCCTATTGCTTAGG | 885 | SIX13 |
| FP6682 | ForcSIX13_ORFr | CACTGTAACTCGGCATCGATG |  |  |
| FP2420 | FEM1P-F1 | AACCCGGCTGGTAGTAATAC | 826 | RFP |
| FP5327 | Tsix1 Rev | AGGGTAAATGAATGGCTGTG |  |  |
| FP2173 | SIX10F | AAAAAGCAGGCTccATGAAGCTCTTGTGGTTG | 520 | SIX10 |
| FP2174 | SIX10R | AGAAAGCTGGGTcCTACTTAGACCTGGTAATTGTT |  |  |
| FP3846 | MdS116_SIX12R | AAAAGCGGCCGCTCAGGAGTGGCATAGCTTGG | 343 | SIX12 |
| FP6447 | SIX12 F WJ | AAAAAGCAGGCTTCACCATGCTGCTCCAAGTACAACCTA |  |  |
| FP1492 | SIX7-F1 | CATCTTTTCGCCGACTTGGT | 862 | SIX7 |
| FP1493 | SIX7-R1 | CTTAGCACCCTTGAGTAACT |  |  |
